# Single cell dynamic phenotyping

**DOI:** 10.1038/srep34785

**Published:** 2016-10-06

**Authors:** Katherin Patsch, Chi-Li Chiu, Mark Engeln, David B. Agus, Parag Mallick, Shannon M. Mumenthaler, Daniel Ruderman

**Affiliations:** 1 Lawrence J. Ellison Institute for Transformative Medicine, University of Southern California, Los Angeles, California, USA; 2 Department of Radiology, Canary Center at Stanford for Cancer Early Detection, Stanford University, Palo Alto, CA, USA

## Abstract

Live cell imaging has improved our ability to measure phenotypic heterogeneity. However, bottlenecks in imaging and image processing often make it difficult to differentiate interesting biological behavior from technical artifact. Thus there is a need for new methods that improve data quality without sacrificing throughput. Here we present a 3-step workflow to improve dynamic phenotype measurements of heterogeneous cell populations. We provide guidelines for image acquisition, phenotype tracking, and data filtering to remove erroneous cell tracks using the novel *Tracking Aberration Measure* (*TrAM*). Our workflow is broadly applicable across imaging platforms and analysis software. By applying this workflow to cancer cell assays, we reduced aberrant cell track prevalence from 17% to 2%. The cost of this improvement was removing 15% of the well-tracked cells. This enabled detection of significant motility differences between cell lines. Similarly, we avoided detecting a false change in translocation kinetics by eliminating the true cause: varied proportions of unresponsive cells. Finally, by systematically seeking heterogeneous behaviors, we detected subpopulations that otherwise could have been missed, including early apoptotic events and pre-mitotic cells. We provide optimized protocols for specific applications and step-by-step guidelines for adapting them to a variety of biological systems.

Advances in live cell imaging and fluorescence time-lapse microscopy have revealed extensive cell-to-cell variation in the spatio-temporal dynamics of critical signaling molecules^1^. This variation has also been shown to drive isogenic cells to exhibit wide heterogeneity in diverse aspects of their phenotype^2^,^3^. Examples include variations in gene-expression^4^,^5^, motility^6^,^7^, morphology^8^,^9^,^10^,^11^, responsiveness to chemical perturbations^12^ and to changes in their microenvironment^3^.

High throughput imaging platforms have recently emerged to enable the study of cell-to-cell phenotypic heterogeneity across large numbers of cells^13^,^14^,^15^,^16^,^17^,^18^,^19^,^20^. These platforms have been broadly impactful for looking at changes in cell growth and viability in response to therapeutic perturbation. However, persistent bottlenecks in imaging and image processing have made it difficult to use these platforms for studying more subtle and dynamic aspects of the cellular phenotype, e.g. monitoring the dynamic interplay between receptor trafficking and cell motility. To enable detailed study of the dynamic phenotype, researchers have developed many computational methods for automated cell segmentation and temporal tracking^21^,^22^,^23^,^24^,^25^,^26^,^27^,^28^,^29^,^30^,^31^,^32^,^33^. Multiple attempts have been made to objectively compare the performance of these diverse tools^34^,^35^,^36^,^37^. Unfortunately, even the best algorithms can fail to track a cell’s phenotype over long time periods, particularly highly motile cells. In addition, experimental design and data collection can impact both cell behavior (e.g. phototoxicity) and tracking. Typically, these tracking failures present themselves with sudden and large changes in phenotypic features such as cell position and morphology. An approach was recently developed to identify erroneous *E. coli* tracks based on unrealistically sudden changes in cell length^33^. In more heterogeneous cell populations, e.g. cancer, researchers are aiming to examine the link between multiple molecular and phenotypic spatio-temporal dynamics. Consequently, there remains a need for methods that consider multiple variables to distinguish between variation and technical artifact.

We have developed a workflow to track the dynamic phenotype of thousands of single cells in heterogeneous populations over short and long time periods. Building upon a high throughput image acquisition platform, our workflow includes optimization of data collection by providing guidelines for the following three steps: (1) image acquisition, (2) phenotype tracking, and (3) data filtering based on the newly defined *Tracking Aberration Measure* (*TrAM,* τ) (Fig. 1). TrAM identifies and enables filtering out unrealistic trajectories to increase data quality. We compute τ based on a combination of cell features that are not essential read-outs of the respective assay to avoid bias. Applied to single cell tracking data, we achieved maximized balanced accuracy across 3 live cell imaging software applications and demonstrate the impact of TrAM filtering on estimations of cellular heterogeneity. We then demonstrate broad biological applicability by measuring cell-to-cell variation of both rapid and delayed time-dependent cell responses, including ligand-mediated receptor activation, cell division and apoptosis (Fig. 1c).

Our data suggest that this workflow can be applied to a variety of biological systems and cell tracking data to more accurately measure the cellular heterogeneity of dynamic phenotypes.

## Results

Cellular dynamics occur on multiple timescales. Consequently, it is important during image acquisition to balance the goal of obtaining precise, high-resolution measurements of dynamical processes while minimizing photobleaching and phototoxicity. During image analysis, one has to consider the trade-off between sensitivity and specificity in detecting invalid tracks for optimization of phenotype tracking. Here, we introduce a workflow to resolve processes that span minutes (e.g. cell motility and nuclear translocation) to hours (e.g. cell proliferation and apoptosis).

### Single cell dynamic phenotyping – overview of the workflow

**Figure 1:**
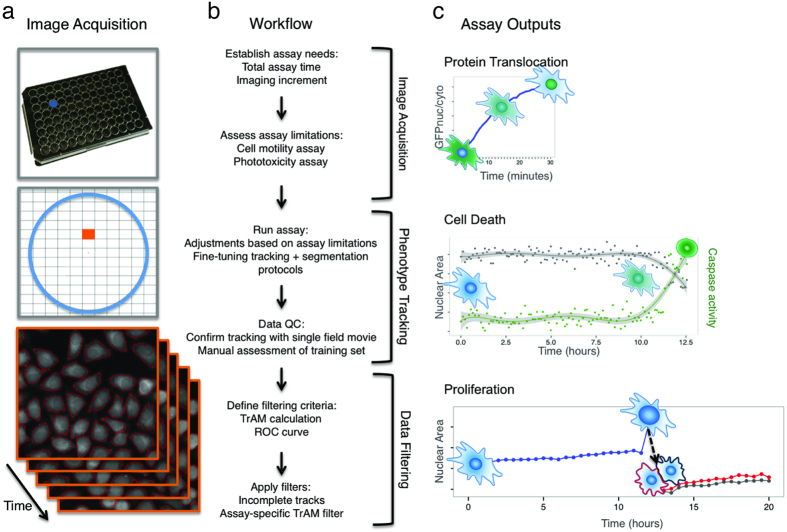
Overview of the workflow. (**a**) Experimental setup and image acquisition. *Top:* Cells were seeded onto 96-well plates, allowed to adhere overnight and imaged the next day. Parallel acquisition of multiple wells allowed for analysis of different experimental conditions including controls. *Middle:* Multiple fields per well (indicated as grid) increased throughput and enabled technical replicates. *Bottom:* Time-lapse imaging and generation of initial tracking data. (**b**) Steps of the workflow including image acquisition, phenotype tracking, and data filtering. (**c**) Examples of assay outputs we measured to track heterogeneity of cellular dynamics. Imaging increment was set according to the expected timescale of response. *Top:* Protein translocation of ligand-stimulated GFP-tagged receptors. Cells were imaged every minute for 30 min, nuclear to cytoplasmic ratios of GFP intensity (GFP_nuc/cyto_) of single cells were plotted over time. Representative cell with GFP translocating to the nucleus is depicted at baseline, intermediate and final time points. *Middle:* Cell death assays. Cells were imaged every 5 min for 5–12 h and nuclear area and caspase activity of single cells were measured over time. Representative cell’s nucleus condensing in the second half of the experiment and initiating effector caspases in response to phototoxic imaging conditions is depicted at baseline, intermediate and final time points. *Bottom:* Cell proliferation assays. Cells were imaged every 30 min for 20 h and mitosis in single cells was tracked over time. Representative cell dividing to 2 daughter cells is depicted to highlight multi-generation tracking.

Our workflow includes 3 steps: (1) image acquisition, (2) phenotype tracking, and (3) data filtering (Fig. 1b). For step 1, total assay time and imaging increments are determined to resolve the desired phenotype. Settings may need to be adjusted based on cell motility and to avoid phototoxicity. For step 2, tracking and segmentation protocols are fine-tuned by cell line and time scale using training data. Quality controls include tracking verification based on manual annotation of single cell tracks. For step 3, tracking data are subjected to downstream filtering to increase data accuracy. This is achieved by eliminating incomplete and aberrant tracks.

Aberrant track removal serves to exclude tracking failures, which often lead to temporal discontinuities in the time series and poor data quality. Therefore, we introduce TrAM (τ), which provides a value calculated for each cell to determine the credibility of its tracking. We adjusted assay-specific TrAM filter thresholds using ground truth Receiver Operating Characteristic (ROC) curves. This enables one to balance the trade-off between sensitivity and specificity in rejecting failed tracking. Step-by-step instructions to calculate and apply TrAM along with the complete source code can be found in the Methods section. We describe below the details of this workflow and demonstrate its application to numerous critical challenges in cellular analysis. We provide optimized protocols for these specific applications and guidelines for adapting them to a variety of assays.

### Cell motility assays – benchmarking and validation

Single cell motility studies can provide valuable insights into cancer cell migration, particularly in the context of cell-cell and cell-environment interactions^6^,^7^. It can also be a useful read-out to determine necessary imaging increments for adequate tracking. Here we describe the steps and analyses we took to increase the precision of cell motility measurements, which include image acquisition, phenotype tracking, and introduction and benchmarking of our novel filtering step based on TrAM.

#### Image Acquisition

The primary variables to be optimized in an image acquisition protocol are cell labeling, excitation/emission spectrum, number of acquisitions, and total acquisition time. When capturing dynamics of cell populations one must choose imaging time increments that will (1) temporally resolve the dynamic phenotype and (2) successfully track as many cells as possible throughout the entire time course. We first conducted preliminary experiments to collect training data sets in which we imaged three cancer cell lines, PC3, Panc-1 and HeLa, every minute for 30 min to determine each cell type’s maximum cell speed. Since nuclei are about 10 μm in diameter and studies report migration rates of <1 μm/min^38^, we can safely assume that even the fastest cell would be adequately sampled under these conditions.

#### Phenotype Tracking

We used the training data sets to develop a base protocol for segmenting and tracking nuclei using the nuclear stains. This base protocol is adaptable to various cell lines and assay time scales (Supplementary Table S1). To adjust the protocol to different cell lines, we set the nuclear diameter to the average measured in 10 cells (HeLa = 20 nm, PC3 = 16 nm and for Panc-1 = 11 nm) and adjusted the splitting coefficient according to their ability to cluster (HeLa < PC3 < Panc-1). Next, we adapted them to different time scales by fine-tuning the range for nuclear area at first and last time points of the experiment. This resulted in more permissive values for long-term time-lapse experiments to include small, apoptotic cells and mitosis, compared to short-term time-lapse experiments of a single cell generation. We removed objects crossing the border of the imaging field to ensure only whole nuclei were analyzed, as they can lead to false morphology assessments.

#### Data Filtering

Once tracking data were collected, we applied a set of filters to increase data accuracy by excluding poor tracks. In our first filtering step, incomplete tracks (i.e. cells without tracking data at every time point) were removed from the dataset. Manual assessment of these excluded cells revealed they were mostly floating debris traversing the focal plane, though some were cells entering or exiting the imaging field. They constituted 13% of the Panc-1 cells, 31% of the HeLa cells, and 42% of the PC3 cells (examples shown in Supplementary Fig. S1). Their removal was thus an important step to ensure the data reflected the viable cell population.

We then applied TrAM filtering to detect cells with segmentation or tracking errors. We used the area and roundness of each cell’s nucleus to calculate TrAM (τ, described in detail in the Methods section). Briefly, TrAM detects changes in tracked values that are sudden, simultaneous, and unusually large. These events typically indicate poor segmentation of cell compartments or failed tracking. TrAM is scaled relative to median fluctuations in tracked values across a cell population, and so is a measure of a cell’s deviation from usual dynamics. Larger values imply more aberrant tracks, with a value of order 1 being typical for the population.

**Figure 2:**
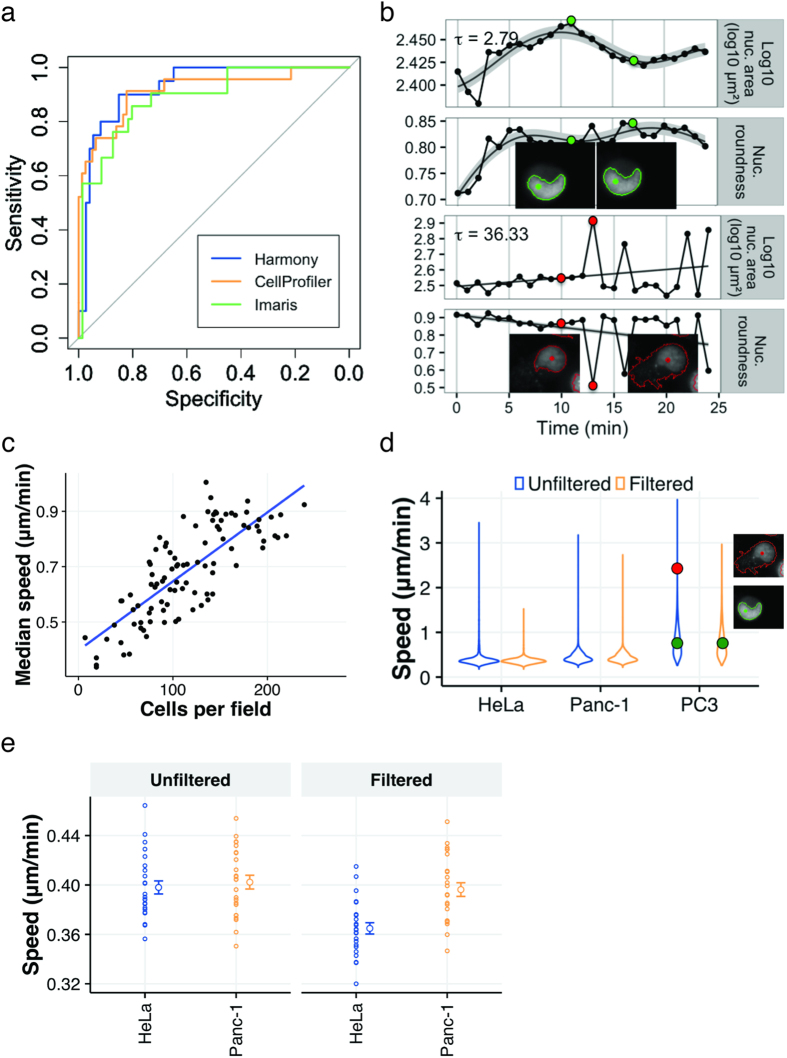
Tracking individual cells in heterogeneous populations. (**a**) Comparison of ROC curves based on data from Harmony AUC = 0.93, CI_95%_ = [0.87,0.98] (blue), CellProfiler, AUC = 0.92, CI_95%_ = [0.84, 0.99] (yellow) and Imaris, AUC = 0.89 95%CI = [0.81–0.97] (green). (**b**) Cell not rejected due to TrAM outlined in green (τ = 2.79). Below outlined in red, cell excluded from evaluation due to TrAM value > 4.81 threshold (τ = 36.33). Nuclear area and roundness are plotted over time. Smoothed curves represent typical fluctuation between adjacent time points. Cell segmentation images correspond to highlighted data points. (**c**) Adjustment of PC3 cell motility to high density imaging areas (R2 = 0.66). Plot correlates number of cells per imaging field with current speed. Each dot represents an imaging well of a 96-well plate. (**d**) Speed distribution of PC3, HeLa and Panc-1 cells before (blue) and after (yellow) TrAM filtering. PC3 data points indicate average speed of examples of high and low τ shown in **b**. (**e**) Speed distribution of HeLa (n = 26 fields, SEM = 0.005) vs. Panc-1 (6 fields, SEM = 0.006) cells post-filtering, p < 0.0007 (*right panel*). In comparison, speed distribution pre-filtering, HeLa SEM = 0.005, Panc-1 SEM = 0.006, p < 0.58 (2-sided t-test) (*left panel*).

To determine a threshold τ value above which we discard tracks as aberrant, we manually annotated 94 randomly selected tracked cells as either pass or fail (Supplementary Data S1). We generated an ROC curve from these annotations and chose the threshold that maximized the sum of sensitivity and specificity. This optimum was at τ = 4.69 (Fig. 2a, Supplementary Movie S1). This filtering step resulted in the exclusion of 8% of the total tracked population of Panc-1 cells, 20% of the HeLa cells, and 23% of the PC3 cells. Filtering increased the precision (data percentage consisting of good tracks) from 87% to 98%. The cost of this improvement was filtering out 15% of the well-tracked cells (85% recall).

An example rejected cell (τ = 36.33) is depicted in Fig. 2b. Note that trajectories of nuclear morphology, specifically area and roundness, contain measurements well outside the curve, which represents credible ranges of the smoothed time series. Snapshots of the cell at these time points confirm poor nuclear segmentation responsible for the jumps. In contrast, a cell typical of those that pass the filtering (τ = 2.79) is depicted above; it is tracked accurately.

To show that TrAM filtering is applicable across multiple image analysis applications, we used the same images to generate tracking data from the surface-tracking algorithm of Imaris (version 8.3.1,) and the open-source software CellProfiler (Analyst 2.0) (Supplementary Movies S2 + 3). We manually annotated a large number of randomly selected tracked cells (91 in Imaris, 102 in CellProfiler) as either pass or fail (Supplementary Data S1). ROC curves from extracted cell tracking data are shown in Fig. 2a and in more detail in Supplementary Figure S2. For Imaris, TrAM filtering increased the precision from 80% to 95%, at 86% recall. For CellProfiler, precision increased from 75% to 97%, at a recall of 82%. Thus, for all three analysis packages, a large improvement in data quality was achieved with a modest cost in throughput. In all of our assays, we chose a threshold that maximized balanced accuracy (the average of sensitivity and specificity). The ROC curve can also be used to adjust the threshold based on other desired trade-offs between data quality and throughput (Supplementary Figure S3).

#### Evaluation

Using the tracking data, we analyzed the heterogeneity of motility within the three cancer cell lines. Cell density has been reported to significantly impact speed^38^. We measured increased average cell speed in higher density fields of PC3 cells (p < 2.2e-16, R^2^ = 0.62) (Fig. 2c), and we observed the opposite effect in Panc-1 cells (p = 0.005, R^2^ = 0.31) (Supplementary Fig. 4). These results emphasize the importance of taking the experimental conditions into account and possible field-to-field fluctuations when evaluating cell motility.

Next, we analyzed the effect of TrAM filtering on evaluations of cell speed in PC3 cells in more detail. Figure 2d demonstrates how filtering reduced the range of measured speed to estimate heterogeneity of the population. The maximum detected speed decreased from 3.97 to 2.96 μm/min. TrAM filtering rejected the poor track in Fig. 2b, whereas it accepted the smooth one. Thus our filtering pipeline can eliminate false outliers of high speed measurements caused by segmentation errors that bias estimates of population heterogeneity.

The importance of incorporating TrAM data filtering into the workflow becomes clear when comparing the motility of multiple cell lines. While the unfiltered data suggested all cell types to have comparable speed maxima, filtering revealed a different picture: HeLa cells were within close proximity of the mean (HeLa decreased from 3.45 to 1.52 μm/min). Although data filtering also reduced the maximum speed measured in Panc-1 (3.18 to 2.73 μm/min) and in PC3 cells, the final population’s speed remained more heterogeneous, including cells well above the mean (Fig. 2d).

Analysis of the filtered data further demonstrated that Panc-1 cells were on average faster than HeLa cells (Panc-1 0.40 μm/min, SEM 0.006 vs. HeLa 0.36 μm/min, SEM 0.005, p = 0.0007, 2-sided t-test) (Fig. 2e). These findings agree with the ground truth established by manual assessment of 166 randomly selected, well-tracked cells across 6 fields (Panc-1 0.39 μm/min, SEM 0.009 vs. HeLa 0.35, SEM 0.007, p = 0.0007, 2-sided t-test). This shows that TrAM’s removing an estimated 15% of well-tracked cells did not bias the result. We would not have detected a significant speed difference without data filtering (HeLa 0.42 μm/min, SD 0.006 vs. Panc-1 0.42 μm/min, SEM 0.006, p = 0.92, 2-sided t-test). Further details on benchmarking and validation are in the Methods section and Supplementary Information. These results demonstrate that TrAM filtering can impact population average and alter the conclusions made from cellular heterogeneity studies.

### Protein Dynamics across the Nuclear Membrane

The dynamics of nuclear translocation can be a powerful read-out to detect heterogeneous responses to various stimuli, for instance sensitivity of a transcription factor to ligand activation or drug inhibition^39^. Here, we apply our method to increase the sensitivity in capturing different levels of heterogeneity of androgen receptor (AR) translocation dynamics in different cell lines.

#### Image Acquisition

In the absence of ligand, AR is located primarily in the cell cytoplasm; however, time-lapse experiments imaging cells every 10 min has revealed AR translocation to the nucleus within minutes after ligand stimulation^39^,^40^. To achieve a higher temporal resolution of the kinetics, we conducted 30 min time-lapse experiments, imaging every 1 min, of cells expressing GFP-tagged AR treated with the agonist R1881.

#### Phenotype Tracking

Quantitative measurements of AR localization require segmentation and tracking of cell compartments. Therefore we used the nuclear stain DRAQ5. Since DRAQ5 signal intensity is low in the cytoplasm, we chose an algorithm for nuclear membrane segmentation that was specifically developed for objects where intensity decreases with distance from the nucleus (Method D in Harmony). Resizing of these regions created a buffer region, which allowed for robust measurements of mean GFP-AR fluorescence in the nucleus and cytoplasm (Supplementary Tables S1 + 2, Supplementary Fig. S5).

**Figure 3:**
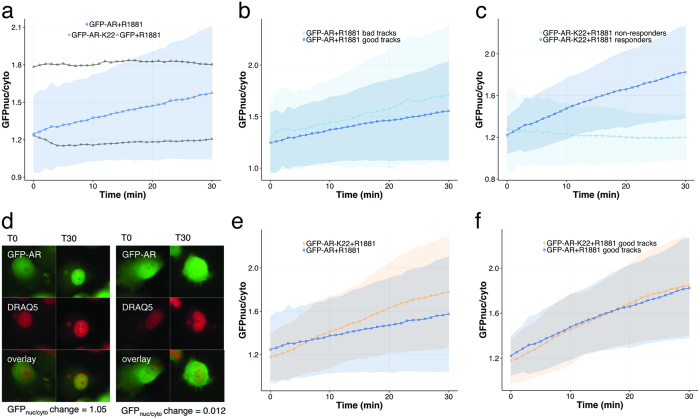
Dynamic measurements of ligand-stimulated protein translocation. (**a**) Nuclear to cytoplasmic GFP intensities in PC3 GFP-AR cells plotted over time. Controls include a non-responsive PC3-GFP cell population (black triangles) and mock-treated cells (black circles). Shaded regions correspond to one standard deviation. (**b**) Effect of 2-step filtering (incomplete tracks + τ) to exclude erroneous cell tracks: bad tracks vs. good tracks. (**c**) Responders vs. non-responders with nuclear to cytoplasmic GFP intensity change < 0.147. (**d**) *Left panel:* Example of responding cell not rejected due to GFP_nuc/cyto_ change > 0.147 (1.05). Snapshots of GFP-AR, nuclear DRAQ5 and overlay at baseline (T0) and at endpoint (T30). *Right panel:* Comparison cell rejected due to GFP_nuc/cyto_ change 0.012. (**e + f**) Effect of filtering on AR translocation kinetics of clonal (yellow) vs. polyclonal (blue) cell lines. Nuclear to cytoplasmic GFP intensities plotted over time. (**e**) Unfiltered data: 0.60 GFP_nuc/cyto_ increase in K22 vs. 0.33 in polyclonal cells, 95% CI of [0.21,0.33], p < 2e-16, first order linear model). (**f**) Filtered data: 0.67 GFP_nuc/cyto_ increase in K22 vs. 0.60 in polyclonal cells, 95% CI of [−0.01, 0.14], p = 0.11, first order linear model).

To obtain quantitative measurements of AR translocation, we calculated nuclear to cytoplasmic GFP intensity ratios in each individual cell at each time point. We applied our method to PC3 cells expressing GFP-AR. We observed nuclear translocation within minutes in these cells, whereas no significant localization change was seen in mock-treated or in GFP-expressing control cells (Fig. 3a).

#### Data Filtering

Next, we applied our 2-step filtering process to exclude erroneous cell tracks. Cells not captured at every time point in the experiment do not inform on the kinetics of this process. Therefore we applied filtering to remove (1) incomplete tracks and (2) cells with poor position tracking. For the latter, we used the X and Y coordinates of each cell’s nucleus to calculate TrAM (τ as described in the Methods section. We generated an ROC curve from manual annotations of pass or fail (Supplementary Data S1), and chose the threshold that maximized the sum of sensitivity and specificity. This optimum was at τ = 2.68 (Supplementary Figure S2).

Figure 3b illustrates good vs. bad tracks based on TrAM, and the filtering effect on the dynamic range of AR localization along with the overall AR trajectory over the time course. Cells expressing GFP-AR displayed notable levels of heterogeneity at baseline (before treatment) and throughout the 30 min time course.

#### Evaluation

We asked whether the level of heterogeneity was exclusively due to genomic variation within the polyclonal cell population. Therefore, we derived a clonal cell line expressing GFP-AR from the population (K22). Since we were interested in comparing the kinetics of AR translocation, we applied an additional filtering step to exclude non-informative cells that did not respond to ligand based on nuclear to cytoplasmic GFP intensity change (GFP_nuc/cyto_). To determine a threshold above which we discard non-responders, we generated an ROC curve based on GFP_nuc/cyto_ intensity change measured in the clonal cell line treated with ligand vs. mock-treated cells (Supplementary Fig. S6). We chose the threshold that maximized the sum of sensitivity and specificity (GFP_nuc/cyto_ intensity change > 0.147, demonstrated in Supplementary Fig. S2). As expected, non-responsive tracks demonstrated a flat line of unchanged GFP_nuc/cyto_ intensity throughout the time course (Fig. 3c). An example non-responder with GFP_nuc/cyto_ change 0.012 is depicted in Fig. 3d (*right panel*). Snapshots of the cell at baseline (T0, before R1881 treatment) and after 30 min treatment (T30) confirm no change in AR subcellular localization. For comparison, a responder with a GFP_nuc/cyto_ change 1.05 is depicted (*left panel*). Snapshots at T0 and T30 of the time-lapse confirm nuclear translocation.

Finally, we compared the curves AR translocation of clonal and polyclonal cell lines. Analysis of unfiltered data suggests that the clonal cell line showed a significantly stronger response, i.e. higher total AR translocation ratio, measured as an increased GFP_nuc/cyto_ change (ΔGFP_nuc/cyto_ change of 0.27 relative to the non-clonal line with a 95% CI of [0.21,0.33], p < 2e-16, first order linear model), demonstrated in Fig. 3e. However, filtered data revealed a different picture: total AR translocation was not significantly different between the cell lines (ΔGFP_nuc/cyto_ change = 0.07 with a 95% CI of [−0.01, 0.14], p = 0.11, first order linear model), demonstrated in Fig. 3f. These findings agree with the ground truth established by manual assessment of 97 randomly selected, well-tracked responding cells (ΔGFP_nuc/cyto_ change = 0.1 with a 95% CI of [−0.07,0.25], p = 0.28, first order linear model) (Supplementary Movies S4 + 5, Supplementary Data S1). In addition, the translocation rates were steady over the time course in both lines (50% translocation after 12.5 min in the clonal line vs. 12.1 min in the polyclonal population). These results are most likely due to a more prominent subpopulation of non-responding cells in the polyclonal cell line (43% vs. 16% in the K22 clonal cell line), rather than differences in the kinetics of AR.

Even after data filtering of non-responders, considerable variation in the trajectories of AR translocation was measured within both cell lines (Fig. 3f), suggesting factors other than genomic variation to be an underlying cause of heterogeneity. AR subcellular localization has been reported to be cell-cycle dependent^41^. However, to our knowledge, the effect of cell cycle on the kinetics of AR has not been elucidated and requires further investigation. Overall, these results demonstrate the ability of our method to measure rapid protein dynamics and quantify contributing subpopulations and translocation kinetics.

### Tracking Nuclear Morphology over extended time periods - evaluation of phototoxic effects

Analysis of nuclear morphology can provide valuable insights into a cell’s state, such as cell cycle phase or cell health^42^,^43^,^44^. Here, we demonstrate how to conduct experiments to track nuclear morphology over extended periods of time and test the limitations of the assay and screen for phototoxic events.

#### Image Acquisition

DRAQ5 is known to induce cytotoxic effects in a time-dependent manner^45^. To detect these effects, we stained nuclei with DRAQ5 and conducted experiments imaging every 5 minutes for 5 hours. Then we asked whether nuclear morphology changes occurred as a result of phototoxicity from extended imaging of DRAQ5 stained nuclei.

#### Phenotype Tracking

For image analysis, we applied protocols to track DRAQ5 stained nuclei with a broader nuclear area range to include apoptotic cells (long assay conditions listed in Supplementary Table S1). Then we extracted tracking data to apply our 2-step filtering process described below.

#### Data Filtering

To obtain reliable tracks of well-segmented nuclei, we calculated τ based on cells’ X, Y coordinates and nuclear roundness using the first 30 time points of the experiment (Supplementary Table S7). While this approach may have failed to identify cells that segmented poorly only in the second half, it ensured that filtering was unbiased toward cells undergoing phototoxicity, which occurred at later time points. Then we used the assay-specific ROC curve shown in Supplementary Figure S2 to determine a threshold of τ = 3.35.

#### Evaluation

**Figure 4:**
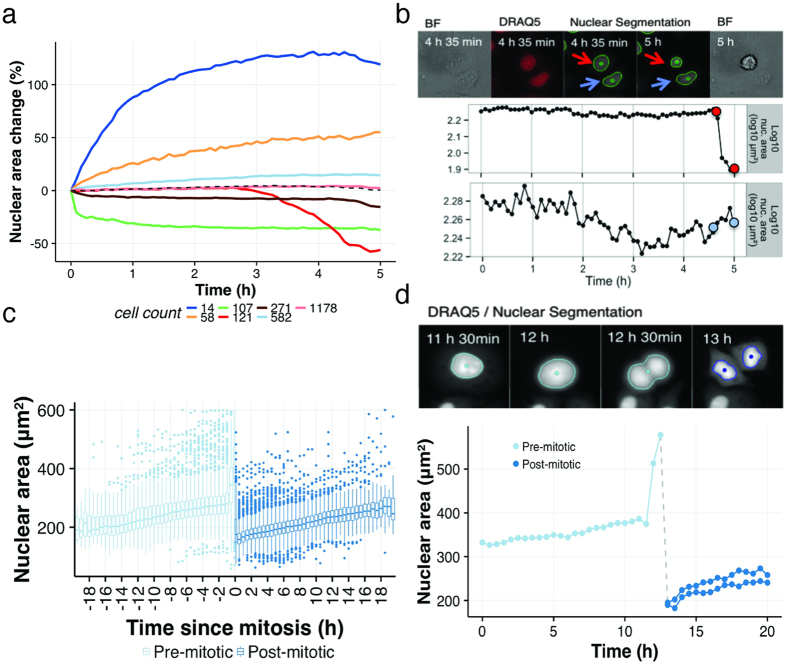
Tracking nuclear morphology. (**a**) K-means clustering of TrAM filtered HeLa cells stained with nuclear DRAQ5 into 7 subpopulations of distinct nuclear morphology trajectories. Dotted line represents population average. (**b**) Example cell with 54% nuclear area decrease due to phototoxic response vs. stable cells. Nuclear areas are plotted over time. Cell segmentation images corresponding to color marked data points depicted on top. Color of arrows and data points indicate the cluster cells fall under in **a**. (**c–e**) Application of TrAM filter threshold τ = 3.71 and cell cycle tracking. (**c**) Box plots of nuclear area normalized to mitotic events detected in Harmony. Nuclei preparing for mitosis depicted in light blue, just born nuclei depicted in dark blue. (**d**) Single cell undergoing mitosis, detected in Harmony and by 37% nuclear area change at T14. Nuclear areas of mother and daughter cells plotted over time. Corresponding cell segmentation images depicted above.

To detect populations of dying cells, we applied K-means clustering to generate subpopulations of distinct nuclear area time courses using *R*’s *kmeans* function with default parameters. Choosing *K *=* 7* clusters, we found a small subset of cells that experienced early signs of phototoxicity (19%), whereas the majority of the total population remained stable throughout the time-course (Fig. 4a). These findings agree with the ground truth established by manual assessment of 100 randomly selected cells, where 17% of the population was identified as early phototoxic events (Supplementary Data S1). The sensitivity of this approach is inherently dependent on the choice of *K*. If the number of clusters is too low, a small or low-population effect may not form its own cluster. If the number is too large, then uninteresting effects unique to particular cells may appear. Because we expected phototoxicity to be evident through shrinking nuclei, we chose the smallest number of clusters (*K *=* 7*) which reliably showed the effect (note that K-means clustering has random initial conditions). In contrast, choosing 5 clusters typically did not reveal the dying population as a separate cluster, and choosing 9 clusters consistently distinguished multiple dying populations (Supplementary Fig. 7). Although the nuclear area change dropped to <50% in dying cells, their small number did not have an effect on the overall population average (Fig. 4a** **+** **Supplementary Fig. 7, dotted lines) and therefore could have been overlooked when analyzing cell images manually.

To illustrate the sensitivity of the assay, Fig. 4b depicts two cells in the same imaging field, one from the red cluster of cells containing phototoxic events (19% of the total population) with a nuclear area decrease of 54% over the time course, and a neighboring cell from the blue cluster of stable cells. These results demonstrate the ability to track nuclear morphology and apply population clustering to identify small subpopulations of cells responding to their environment that might otherwise be missed. Therefore, this approach can be used to identify assay limitations with high sensitivity.

### Tracking Nuclear Morphology over extended time periods – tracking cell division

Nuclear area changes occur not only during apoptosis but also in healthy, proliferating cells in the course of the cell cycle^46^,^47^. Here, we demonstrate how we adjusted image acquisition to avoid the phototoxic effects demonstrated above, and track nuclear morphology to evaluate cell proliferation.

#### Image Acquisition

First, we changed the nuclear marker to Nucleus-RFP, the time increment to 30 min and total number of image acquisitions to T = 40 to avoid the effects of phototoxicity while capturing cell cycle dynamics and mitosis. Based on a HeLa cell motility rate of 0.37 μm/min (22 μm/h) and nuclei of approximately 20 μm in diameter, we were confident that we could effectively track cells by imaging every 30 min for 20 hours without inducing phototoxicity.

#### Phenotype Tracking

For image analysis, we applied the same tracking protocols as described in the **phototoxicity assay** section after adjusting the nuclear identification to the DsRed channel to capture the Nucleus-RFP expressing nuclei (long assay conditions listed in Supplementary Table S1).

To ensure that photoxicity had been eliminated, we applied data filtering and K-means clustering as described previously to generate 3, 5 and 7 clusters of nuclear area time courses (Supplementary Fig. S8). None of these analyses revealed a subpopulation with the >50% nuclear area reduction indicative of phototoxicity. These results confirmed proper adjustment of our image acquisition and phenotype tracking protocols.

#### Data Filtering

Having confirmed optimized image acquisition, we used the tracking data to evaluate mitosis. We set the TrAM filter threshold to τ = 3.41 across the full time-lapse to exclude poor tracks. The cut-off to maximize balanced accuracy was established based on the mitosis-specific ROC curve in Supplementary Figure S2.

#### Evaluation

On average, Harmony found 3.1% of the population dividing into two daughter cells per hour. In contrast, proliferation was almost entirely blocked in the DRAQ5 stained nuclei (0.7% cells dividing per hour, Supplementary Fig. S9), due to the phototoxic imaging conditions and a known effect of the nuclear stain^48^. In agreement with previous reports, we measured a slow, stable increase of nuclear area during interphase, followed by an episode of nuclear swelling during the final hour before cell division (Fig. 4c)^46^,^47^.

To predict mitosis based on nuclear morphology, we used the data set to establish the ground truth by annotating 46 mitotic events and analyzing nuclear area change before mitosis. We set a detection threshold to 18.2% maximum nuclear area change within a single time point (30 min) based on the ROC curve to detect mitotic events in Supplementary Figure S2. We identified 3.1% of the population to be pre-mitotic per hour, confirming Harmony’s tracking of mitotic events. An example is demonstrated in Fig. 4d, where a parental cell (37% maximum nuclear area change at T14) divides to 2 daughter cells, detected by maximum nuclear area increase. These results demonstrate the ability to track nuclear morphology over extended time periods to detect events occurring in small subpopulations of cells, including phototoxicity and mitosis.

In some extended time-lapse experiments, nuclear markers are not available due to low transfection efficiency of nuclear markers and cytotoxic effects of nuclear stains. In these cases, cell morphology features can be used to track cell phenotypes, and are thus key read-outs in drug screening assays^21^,^49^,^50^. Therefore, we adapted the workflow to track cellular phenotypes based on non-toxic Cell Tracker dyes, from the time of drug treatment until detectable response. Details on image acquisition, phenotype tracking and data filtering, including a specific application can be found in the Supplementary Applications section of the Supplementary Information.

## Discussion

There is increasing evidence that cell-to-cell variation contributes to heterogeneity in response to drug treatments. High throughput imaging and automated image analysis approaches have been imperative in advancing our understanding of systems biology and providing a platform to study various aspects of phenotypic heterogeneity. Here, we developed a workflow that leads to improved cell phenotype tracking of heterogeneous populations using high throughput imaging platforms.

Instrumental to this workflow is a post-processing method to filter tracking data based on the novel *Tracking Aberration Measure* (*TrAM*). We used a training data set to establish the ground truth and to apply the ROC approach to objectively select filtering thresholds and maximize the value of tracking data from multiple software applications. Once the filter cut-off is determined, the assay can be run in high throughput without further adjustment. A new ROC curve is only necessary when developing an entirely new experiment. While this requires more effort up-front than simple, qualitative tracking validation, TrAM increases the overall data quality. It also provides metrics for adjusting the filter’s trade-off between precision (the fraction of included cells that are well tracked) and recall (the fraction of well-tracked cells that are included).

The goal of TrAM filtering is to increase data quality without introducing bias by underestimating heterogeneity. It detects unusually large changes occurring simultaneously in multiple tracked quantities. These jumps are assumed more likely due to segmentation errors than to real cellular activity. By verifying that the TrAM ROC curve has an AUC close to 1, we can be fairly certain that few valid tracks are filtered out. If the AUC is instead much lower, the assumption of a good separation between actual and false phenotypes must be re-evaluated. In such cases we suggest adjusting the cut-off to increase specificity, and verifying that the excluded events do not bias the assay results. In Supplementary Figure 3 we highlight the flexibility to favor specificity over sensitivity and vice versa, and demonstrate the impact of applying different cut-offs on throughput and outcome. While we chose to remove cells with poor tracking, one could alternatively devise methods to detect and correct the resulting time series^33^.

Our filtering method led to a better estimate of the mean phenotype and led to different conclusions than unfiltered data. Using unfiltered data, we drew the false conclusion that HeLa and Panc-1 cells are equally motile. These data contained HeLa cells that presented with erroneous jumps in their trajectories, resulting in mistakenly fast cell speeds. Excluding these outliers revealed that the Panc-1 cells in fact migrated at a significantly higher speed than the HeLa cells, matching our assessment using ground truth. It has been previously shown that various experimental conditions, including seeding density, time of cultivation, pH and temperature of the medium significantly impact measurements of cell speed^38^. We confirmed the influence of cell density and measured increased cell speed in higher density fields of PC3 cells and the opposite effect in Panc-1 cells. Thus, ensuring the robustness of cell motility results, especially when comparing different cell lines, requires experiments across culturing conditions such as cell density. When comparing AR translocation of a clonal cell line with a polyclonal population, we found differing average response. However, data filtering revealed that the stronger response in the clonal line was due to a smaller fraction of non-responders contributing to the average effect rather than differing kinetics in responsive cells. When instead restricting just to responsive cells, the average AR translocation change was not significantly different between the two lines. This was borne out in a ground truth analysis, and is not altogether surprising considering the clonal line was derived from the heterogeneous population. Appropriate data filtering to ensure comparison of alike phenotypes is thus important for avoiding false discoveries.

High throughput assay data will inevitably contain two distinct types of variation, noise due to technical artifacts, and cell heterogeneity. K-means clustering of our data enabled the detection of small but relevant subpopulations with distinct behavior. When we applied it to extended DRAQ5 tracking data, we found a cell population that experienced phototoxicity. Because the chosen number of clusters impacts what can be discovered (e.g. Supplementary Figures S7 + 8), we suggest applying multiple K-values for each data set. Furthermore, because of variability due to random initial conditions, we recommend attending only to those K-values for which the results are stable across multiple clustering runs. Finally, after experimental optimization to avoid phototoxicity, we used the ROC approach to detect cells that were preparing to divide into two daughter cells. These small fractions of cells could have been missed when either evaluating images by eye or averaging across the cell population.

We believe the presented workflow will enable scientists to use automated image analysis software for live cell imaging data more effectively. The assays developed here are optimized and can be applied using the detailed protocols we provide. In addition, we include step-by-step instructions that allow researchers to further develop or modify the protocols according to research needs. While we have focused on basic research applications, the workflow may also be useful in translational research to accurately track drug response to gain much needed insight into the dynamical origins of cellular heterogeneity.

## Methods

### Cell culture

#### Culture conditions

PC3, HeLa and Panc-1 cell lines were obtained from ATCC. PC3 and HeLa cells were maintained in RPMI1640 (Corning, cat. no. 10-040), Panc-1 cells in DMEM (Corning, cat. no. 10-013), all supplemented with 10% heat-inactivated GemCell bovine serum (Gemini Bio-Products, cat. no. 100-500), Penicillin-Streptomycin (Gemini Bio-Products, cat. no. 400-109). PC3 cells stably expressing GFP-AR were additionally treated with 200 ng/ml G418 sulfate solution (Gemini Bio-Products, cat. no. 400-113) for positive selection. Cells were maintained at 37 °C in a humidified incubator with 5% carbon dioxide.

#### Generation of PC3 cell line stably expressing GFP-AR

PC3 cells were transfected with the pEGFP-C1-AR plasmid (a gift from Michael Mancini, Addgene plasmid # 28235) using FuGENE HD Transfection reagent (Promega). Selective conditions were applied 48 h later using 300 μg/ml G418 (Gemini, cat. no. 400-113), and media was changed every 48 h for 2 weeks, when stable clones were visibly expanding. EGFP positive cells were selected for by fluorescence-activated cell sorting (FACS) to obtain a heterogeneous GFP-AR expressing cell population. The PC3 GFP-AR K22 clonal line was developed by sorting single GFP positive cells into a 384-well plate and expanding. The PC3 GFP control cell line was generated using MISSION pLKO.1-puro-CMV-TurboGFP Positive Control Transduction Particles, (Sigma Aldrich, cat. no. *SHC003V*) followed by FACS (FACSAria).

### Cell preparations for experiments

#### Cell seeding

Cells were seeded into a 96-well CellCarrier plate (PerkinElmer, cat. no. 6005550) at a density of 5,000–12000 cells/well for cell response assays, and 3000 cell/well to track mitosis, using phenol red free RPMI (Biochrom, cat. no. F1275) media supplemented with charcoal:dextran stripped FBS (Gemini Bio-Products, cat. no. 100-119) and L-Glutamine (Gemini Bio-Products, cat. no. 400-106). All experiments were performed in 100 μl total volume per well.

#### Transient transfections

Transient transfections were performed using 100 ng EGFP-AR and FuGENE (Promega, E2311) and/or 4 μl tubulin-RFP (CellLight Tubulin-RFP, BacMam 2.0 Thermo Fisher Scientific, cat. no. C10614), or 4 μl (CellLight Nucleus-RFP, BacMam 2.0 Thermo Fisher Scientific, cat. no. C10603) per well. Cells were and incubated overnight before experiments were performed.

#### Cell labeling

In experiments using nuclear stain, 2.5 μM DRAQ5 (Abcam, cat. no. ab108410) was added to cells 30 min before starting the experiment. For whole cell labeling, cells were incubated with CellTracker Orange CMTMR Dye at 1:10,000 (Thermo Fisher Scientific, cat. no. C2927) for 30 min. Excess dye was washed away 3x with media directly before experiments were performed. For caspase-activity measurements, cells were loaded with 2 μM CellEvent Caspase-3/7 Green Detection Reagent (Thermo Fisher Scientific, cat. no. C10423) directly before the experiment.

#### Drug treatments

Methyltrienolone (R1881, Perkin Elmer, cat. no. NLP005005MG) and paclitaxel (LC Laboratories, cat. no. P-9600) were pre-dissolved in DMSO to obtain 10 and 20 mM stock solutions, respectively. Staurosporine solution, 1 mM in DMSO, was purchased from Sigma-Aldrich (cat. no. S6942). For cell treatment, working solutions of a 10x concentration of the desired end concentration were prepared using media. In the course of the experiment, cells in 90 μl media were treated with 10 μl working solution to achieve a 100 μl total volume of 50 nM paclitaxel or 250 nM staurosporine for each well.

### Image Acquisition

#### Live cell imaging

Image acquisition was performed using the Operetta High Content Screening (HCS) instrument (PerkinElmer). Cells were imaged with an Olympus LCPLFLN 20 × 0.45 objective in 100 μl total volume. Fluorescence images were acquired using minimal lamp power to reduce phototoxicity and bleaching.

#### Short-term time-lapse experiments

For experiments capturing short-term dynamics, a time series of 30 min at 1 min intervals was set to break after acquisition of a baseline image (T-1), allowing for manual addition of ligand, and immediate image acquisition thereafter (T0-30). Technical (multiple wells per run) and biological (multiple runs per day) replicates were performed. A minimum of 2 fields per well were imaged, depending on the number of wells, channels, and exposure time, to stay within the 1 min interval. Control wells were mock-treated and imaged in parallel to measure ligand independent responses. For protein translocation assays, T0-T30 were evaluated. For cell motility, T6-T30 were analyzed to correct for thermal shift due to the system perturbation.

#### Long-term time-lapse experiments

Drug response experiments were performed for 12 h at 5 min increments, and included a pause in the time series after T-1 for direct drug treatment before proceeding to T0. A minimum of 2 fields per well were imaged, depending on the number of wells, channels, and exposure time, to stay within the 5 min interval. Control wells were mock-treated and imaged in parallel to compare with drug-treated cells. Additional wells were imaged only twice, before and after the time course, to ensure comparable cell morphology and cell density with those wells subjected to frequent light exposure during the time-lapse experiment. For downstream image analysis, T0-T144 were evaluated. Phototoxicity assays were performed imaging cells with nuclear stain DRAQ5 every 5 min for 5 h. For cell cycle and mitosis, Nucleus-RFP expressing cells were imaged and tracked every 30 min for 20 h.

### Phenotype Tracking

#### Nuclear Measurement and Tracking

In the Harmony 3.5.2 software (Perkin Elmer), building block Find Nuclei was used to identify objects stained with DRAQ5 and detected in the far red DRAQ5 channel (excitation 620–640, emission 650–760). Method M was used along with cell-line specific parameters to measure nuclear morphology properties as listed in Supplementary Table S1. Using the Select Population building block, (1) border objects were excluded resulting in Nuclei Selected, and (2) nuclear area measurements were used to select for nuclei within acceptable range. The resulting Final Nuclei were subjected to the Track Objects building block and options (1) Track Object Division, (2) Correct Detection Errors (3) Discard Single Timepoint Tracks were checked and applied to the algorithm. Morphology and Kinetic Properties of the Tracked Final Nuclei were calculated in Harmony. Results were defined and exported using the List of Outputs option to retrieve population based text files containing mean values of selected single cell data. The Objects Population – Tracked Final Nuclei data for cells tracked at 1 min intervals and present at all 25 time points were subjected to TrAM filtering. Therefore, we extracted the morphology features ‘nuclear area’ and ‘nuclear roundness’, which was calculated in Harmony as follows:


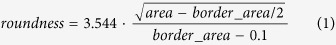


Here, *border_area* represents the perimeter of the nucleus.

In Imaris (version 8.3.1, Bitplane), the Surface Tracking routine was applied to identify objects stained with DRAQ5 and detected in the far-red DRAQ5 channel (excitation 620–640, emission 650–760). The algorithm first detects, and then connects objects to create object trajectories. The surface area detail level was set to 2 μm, and the background subtraction by local contrast for object identification was applied. ‘Split touching objects’ was used with seed point diameters as follows: 5 μm for PC3 and Panc-1 cells, and 10 μm for HeLa cells. The largest object diameter was set to 30 μm for PC3 and Panc-1 and 40 um for HeLa cells; the smallest total voxel for an object was set to 300. To create trajectories, ‘Autoregressive motion’ mode was used with maximum moving distance between frames set to 5 μm. No gap (i.e. object ‘skip’ one or more frames) was allowed. For downstream TrAM filtering of full-length tracks, we extracted the morphology features ‘Area’ for nuclear area and ‘Oblate Ellipsoid’ for nuclear roundness, calculated as follows:


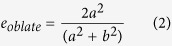


Here, *a* and *b* represent the lengths of the semi-axes of the nucleus.

In CellProfiler (version 2.1.1), cell nuclei were identified using the IdentifyPrimaryObjects analysis module, then tracked using the TrackObjects module. MeasureObjectIntensity and MeasureObjectSizeShape were used to determine the brightness and shape of nuclei. The full setup can be found in the Supplementary Information. For downstream TrAM filtering of full-length tracks, we extracted the morphology features ‘Area Shape’ for nuclear area and ‘Form Factor’ for nuclear roundness, calculated as follows:


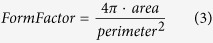


#### Protein dynamics across the nuclear membrane

In addition to the Harmony building blocks described above, the DRAQ5 channel was used to Find Cytoplasm of the Tracked Final Nuclei population using Method D and adjusting the individual threshold according to the fluorescent signal intensity of the respective cell line (listed in Table 3 of the Results section). Nuclear and Cytoplasm Regions were selected and resized by adjusting inner and outer boarders as listed in Supplementary Table S2, and Mean EGFP Intensity Properties were calculated using these regions. To measure protein translocation, the Nuclear to Cytoplasm (N to C) Ratio was measured in every cell at each imaging time point. This value was generated with the Harmony building block Calculate Properties by Formula a/b, and defining “a” as Intensity Nucleus Region EGFP Mean and “b” as Intensity Cytoplasm Region EGFP Mean. A subsequent step was integrated to Flag Responders having values 0.9–10. Results were defined and exported using the List of Outputs option to retrieve population based text files containing mean values of selected single cell data and max values of Responders 0.9–10. Full trajectories were subjected to TrAM filtering.

#### Tracking cell morphology changes in response to drugs

For extended time-lapse experiments, instead of measuring and tracking nuclei, building block Find Cells was used to identify objects in the dsRed channel (excitation 520–500 nm, emission 560–630 nm), or alternatively for a label-free option, in the digital phase contrast (DPC) channel using the High Detail Mode. In both cases, Method C was used along with cell-line specific parameters to measure cell morphology properties as listed in Supplementary Tables S4. Using the Select Population building block, (1) border objects were excluded resulting in Cells Selected, and (2) cell area measurements were used to select objects within acceptable range. The generated population Cells Selected Final were subjected to the Track Objects building block and the following options applied to the algorithm: (1) Track Object Division, (2) Correct Detection Errors and (3) Discard Single Time point Tracks. Morphology and Kinetic Properties of the Tracked Cells Selected Final were calculated in Harmony. Results were defined and exported using the List of Outputs option to retrieve population based text files containing mean values of selected single cell data. The Objects Population – Tracked Cells Selected Final were subjected to TrAM filtering.

#### Tracking initiation of apoptosis in real time

In addition to tracking cell morphology as described above, the Harmony building block Select Cell Region was applied to generate a Background Region through resizing inner and outer boarders of the ring region as listed in Supplementary Tables S5. Mean EGFP Intensity Properties were calculated for regions (1) cell and (2) background. To measure EGFP signal and apoptosis initiation, the Signal to Background Ratio was measured in every cell at each imaging time point. This value was generated with the Harmony building block Calculate Properties by Formula a/b, and defining “a” as Intensity Cell EGFP Mean and “b” as Intensity Background Region EGFP Mean. A subsequent step was integrated to Flag Cells Selected Dead EGFP by defining a threshold of >1.1 for the GFP Signal to Background Ratio for every cell. This flagging step enables tracking of cell morphology and analysis of kinetic properties in cells during both live and dead stages of the experiment. Results were defined and exported using the List of Outputs option to retrieve population based text files containing mean values of selected single cell data. Trajectories were subjected to TrAM filtering.

#### Global motion correction

Because well plates can expand slightly during thermal equilibration, we corrected for any average cell motion between acquisition time points. We assumed that cells move randomly so there is no net direction of cellular motion. For cells tracked at all time points during acquisition, we used the change in their average (X, Y) position as an estimate of plate motion. This plate motion trajectory was then subtracted from the positions of all tracked cells to provide their corrected positions, which were then used in all subsequent computations.

### Data Filtering

#### Rationale behind the creation of tracking aberration measure (TrAM, τ)

Capturing the spatio-temporal dynamics of cellular phenotypes is a complex process that requires sophisticated cell tracking and filtering algorithms to accurately depict the data. For example, if one wants to increase the time interval between adjacent image acquisitions to reduce phototoxicity, the image-to-image changes can become quite large, and oftentimes fool tracking algorithms. This can result in cell reports with incorrect cell positions, membrane boundaries, and subcellular compartment areas, to name a few. Because these tracking failures result in poor data, we devised an algorithm to detect and remove these cases. By visual inspection we noted that poorly tracked cells tended to have sudden and large changes in feature quantitation over time that was absent in well-tracked cells, which tended to have smooth trajectories. Such changes typically occurred in features such as cell position, area, and average fluorescence. Tracking failures tend to influence multiple measurements; thus, a strong indication of a tracking failure was a simultaneous jump in multiple feature quantities. We therefore devised an algorithm that is sensitive to unusually large changes occurring simultaneously in multiple tracked quantities. We term this value *trajectory aberration measure* (*TrAM*).

### Definition of the 2-step data filtering process

#### Incomplete tracks

Analysis of single cell dynamics requires successful tracking throughout an experiment. In Harmony, cell positions in two consecutive time points must overlap for an object to be tracked. In Imaris and CellProfiler, gaps were set to 0 to obtain most comparable tracking results. After executing a tracking algorithm, a certain percentage of cells was excluded from the data due to incomplete cell tracking (i.e. data missing across consecutive time points for a particular cell ID). For our experiments, the tracking data exported from Harmony included the following percentage of cell IDs not listed at every time point of the experiment: 13% Panc-1, 31% HeLa and 42% PC3.

#### Tracking Aberration Measure

We introduce the *Tracking aberration measure* (*TrAM,* τ), which reflects how far multiple time series deviate from their smoothed time series relative to expectation, with simultaneous deviations emphasized:


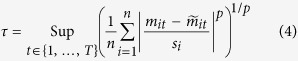


Here *T* is the total number of time points measured, *n* is the number of distinct time series measured, *m*_*it*_ is the *t*^th^ measurement of the *i*^th^ time series, 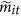 is the smoothed *i*^th^ time series, *s*_i_ is the typical scale of fluctuations in the *i*^th^ time series, and *p* is an exponent which determines how sensitive the measure is to simultaneity of large fluctuations in multiple time series. The aberration reflects the single worst (greatest value) time point in terms of simultaneous fluctuation away from smooth time series. For every time point, each of the *n* time series was assessed for deviation from its smoothed trajectory (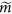) in units of its typical fluctuations (*s*). Because we apply the *p*-norm to these quantities with *p* = 0.5, the aberration is highest when a given amount of total deviation is spread evenly across all time series as opposed to concentrated in only a few time series. This makes the measure sensitive to simultaneous jumps in multiple measures, which is a good indicator of a tracking failure.

When including (X, Y) tracks we modified the formula to ensure it treats all motion directions equally. Since the *p*-norm is anisotropic for p ≠ 2, we modified τ to include a Euclidian term for spatial motion:


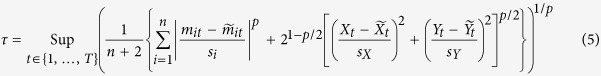


Here *n* is the number of measurement time series in addition to (X,Y) coordinates to be included in the TrAM calculation.

We created the smoothed time series 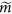 using cubic smoothing splines (via the *R* function *smooth.spline*). The number of knots in the spline should reflect the amount of credible temporal variability in the measurements. For our assays, we chose the number of knots to be 5 for all time series.

We assessed the typical fluctuation scale for a particular measurement, *s*_*i*_, across all cell tracks of a given cell type under a given set of conditions (e.g. treatment). It is the median absolute value of adjacent temporal measurements:


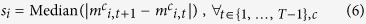


Here 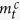 is the measurement at time *t* on cell *c*. By using the median instead of the mean the quantity is less sensitive to large jumps due to the relatively few number of poorly tracked cells and should thus reflect the typical fluctuations in time series of well-tracked cells. A quantile other than the 50% can be used in cases where more or fewer poorly tracked cells are present. Parameters used to calculate TrAM and threshold settings are listed for each assay in the Supplementary Table 7.

The code to calculate TrAM can be downloaded at:

https://github.com/RudermanLab/tram

### Construction of ground truth from manual annotation of cell tracks

To evaluate the performance of our method, it was necessary to establish a ground truth. Therefore, we imaged 3 different cell lines (PC3, Panc-1 and HeLa) every minute for 30 min. We generated tracking data as described in the Online Methods with Harmony (v3.5.2 Perkin Elmer), Imaris (v8.3.1, Bitplane) and CellProfiler (Analyst 2.0). To judge tracking accuracy by eye, we generated animations of the nucleus images used to track overlayed with outlines of nuclear segmentation in each application (Supplementary Movies S1–5). A trained cell biologist (K.P.) examined by eye tracks for randomly chosen cells to judge each as “pass” or “fail” (Supplementary Data S1). These annotations constituted the ground truth against which our algorithmic approach to detecting tracking failures was compared.

### Generation of Receiver Operating Characteristic (ROC) curves

To objectively determine a threshold for our TrAM filters we applied the ROC formalism. We chose the threshold that maximizes the Youden Index^51^, which is equivalent to maximizing balanced accuracy (the average of sensitivity and specificity). We generated an ROC curve for each application’s tracking using the *R* package *pROC* by associating the ground truth for each cell’s tracking (“pass” or “fail”, listed in the columns nuc.by.eye.ROC in Supplementary Data S1) with the τ computed for that track. Supplementary Figure 2a–c shows the resulting ROC curves for comparison across applications. Harmony (τ threshold of 4.69) resulted in 90% sensitivity and 85% specificity to detect failed tracks. Imaris (τ threshold 2.27) had sensitivity of 86% and specificity of 80%. CellProfiler (τ threshold 2.84) had 91% sensitivity and 82% specificity. These high sensitivities and specificities indicate that TrAM filtering can eliminate a large proportion of failed tracks while dismissing a small fraction of good data. Note that other criteria and measures for choosing thresholds may be more appropriate (e.g. positive predictive value, accuracy), depending on the research goals.

#### Threshold Selection for Additional Applications

For the protein translocation assay, we used cells’ X, Y values obtained from Harmony to calculate τ. Using an ROC curve based on 107 cells (75 pass/32 fail, listed in column XY.by.eye.ROC of Supplementary Data S1) we selected 2.68 as the maximum TrAM cut-off for valid tracking by Harmony. This achieved 94% sensitivity and 96% specificity (Supplementary Fig. 2d). For the phototoxicity assay, we assessed TrAM using X, Y and nuclear roundness of time points T0-T30. The ROC curve was generated using 100 randomly selected cells (61 pass/39 fail, listed in column by.eye.ROC of Supplementary Data S1), with a selected cut-off τ of 3.35. This tracking failure classifier had 87% sensitivity and 92% specificity (Supplementary Fig. 2e). For the mitosis assay, we included X, Y and nuclear roundness of the full time courses to calculate τ. Using an ROC curve based on 90 cells (66 pass/24 fail, listed in column by.eye.ROC in Supplementary Data S1) we selected 3.41 as the maximum τ cut-off for valid tracking by Harmony. This achieved 83% sensitivity and 94% specificity (Supplementary Fig. 2f).

### Benchmarking and Validation

#### Efficiency of failed tracking removal using TrAM

The goal of TrAM is to improve data quality by removing cells whose tracking has failed while preserving well-tracked cells. The inclusion of poorly tracked cells adds both bias and variance to the data, whereas excluding well-tracked cells reduces *n*, leading to increased statistical uncertainty. To quantify this impact, we used the Precision-Recall framework. For us, precision quantifies the fraction of accepted cells whose tracking was accurate, and recall is the fraction of accurate cells that are accepted in the data set. These quantities can be estimated from the operating point on the ROC curve if the prevalence of failed cell tracks is known.

We estimated tracking failure prevalence as the fraction of randomly selected cells evaluated by eye, which did not pass visual inspection. This was done for Harmony, Imaris and CellProfiler and listed as “pass” or “fail” in the column nuc.by.eye.total in Supplementary Data S1. Precision without filtering is 1-Prevalence. With TrAM filtering, the Precision is given by:


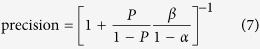


Here *P* is the failed tracking prevalence in the cell population, α is 1-Specificity, and β is 1-Sensitivity. Sensitivity and Specificity were drawn from the operating point of the ROC curve.

For Harmony, we achieved a precision of 98% up from a pre-filtering value of 83%. The cost of this improvement was a recall of 85%, thereby losing 15% of well-tracked cells. For Imaris, the precision increased from 80% to 95%, at 86% recall. For CellProfiler, precision increased from 78% to 97%, at a recall of 82%. Thus in call cases a great improvement in data quality was achieved with a modest cost in throughput. By adjusting the TrAM threshold these trade-offs can be adjusted to favor either data quality or throughput.

Additional assay-specific validation points are found in the Supplementary Information.

### Statistical Analysis

For data filtering and statistical analyses, we used R version 3.3.0. We used the function *kmeans* with default settings to perform k-means clustering.

## Additional Information

**How to cite this article**: Patsch, K. *et al*. Single cell dynamic phenotyping. *Sci. Rep.* **6**, 34785; doi: 10.1038/srep34785 (2016).

## Supplementary Material

Supplementary Movie S1

Supplementary Movie S2

Supplementary Movie S3

Supplementary Movie S4

Supplementary Movie S5

Supplementary Information

Supplementary Dataset 1
