# Supplementary material for: Single cell dynamic phenotyping
Source: Sci Rep. 2016 Oct 6;6:34785. doi: 10.1038/srep34785 (PMC5052535; doi:10.1038/srep34785)
Supplement: Supplementary Information [file srep34785-s6.pdf]

Supplementary Information

Single cell dynamic phenotyping

Authors:

Katherin Patsch[1], Chi-Li Chiu[1], Mark Engeln[1], David B Agus[1], Parag Mallick[2], Shannon M. Mumenthaler[1], Daniel Ruderman[1]*

Affiliations:

1: Lawrence J. Ellison Institute for Transformative Medicine, University of Southern California, Los Angeles, California, USA;

2: Department of Radiology, Stanford University, Stanford, CA, USA;

*Correspondence should be addressed to D.R. (ruderman@usc.edu)

SUPPLEMENTARY FIGURES

Filtering Step 1: Incomplete cell tracks

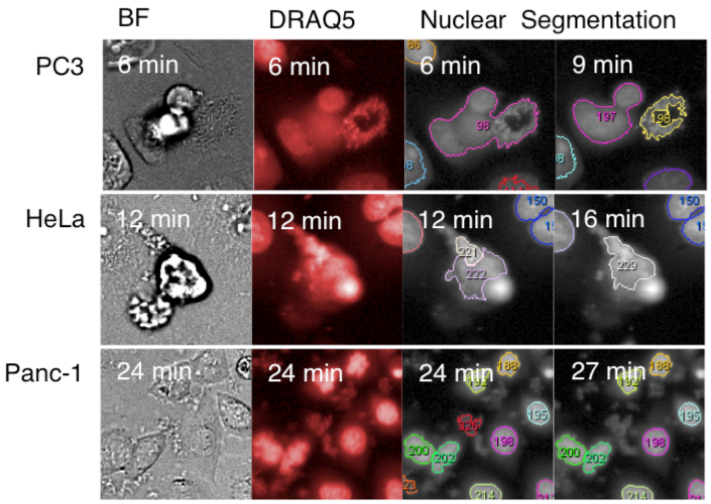

Supplementary Figure S1: Examples of incomplete cell tracks filtered and excluded from further analyses. PC3 cell ID 98 detected at 6 min, HeLa cell ID 222 at 12 min and Panc-1 cell ID 326 at 24 min, are not detected minutes later. BF (brightfield image), DRAQ5 nuclear segmentation image, Cell ID overlay with DRAQ5 in gray.

Receiver operating characteristic (ROC) curves

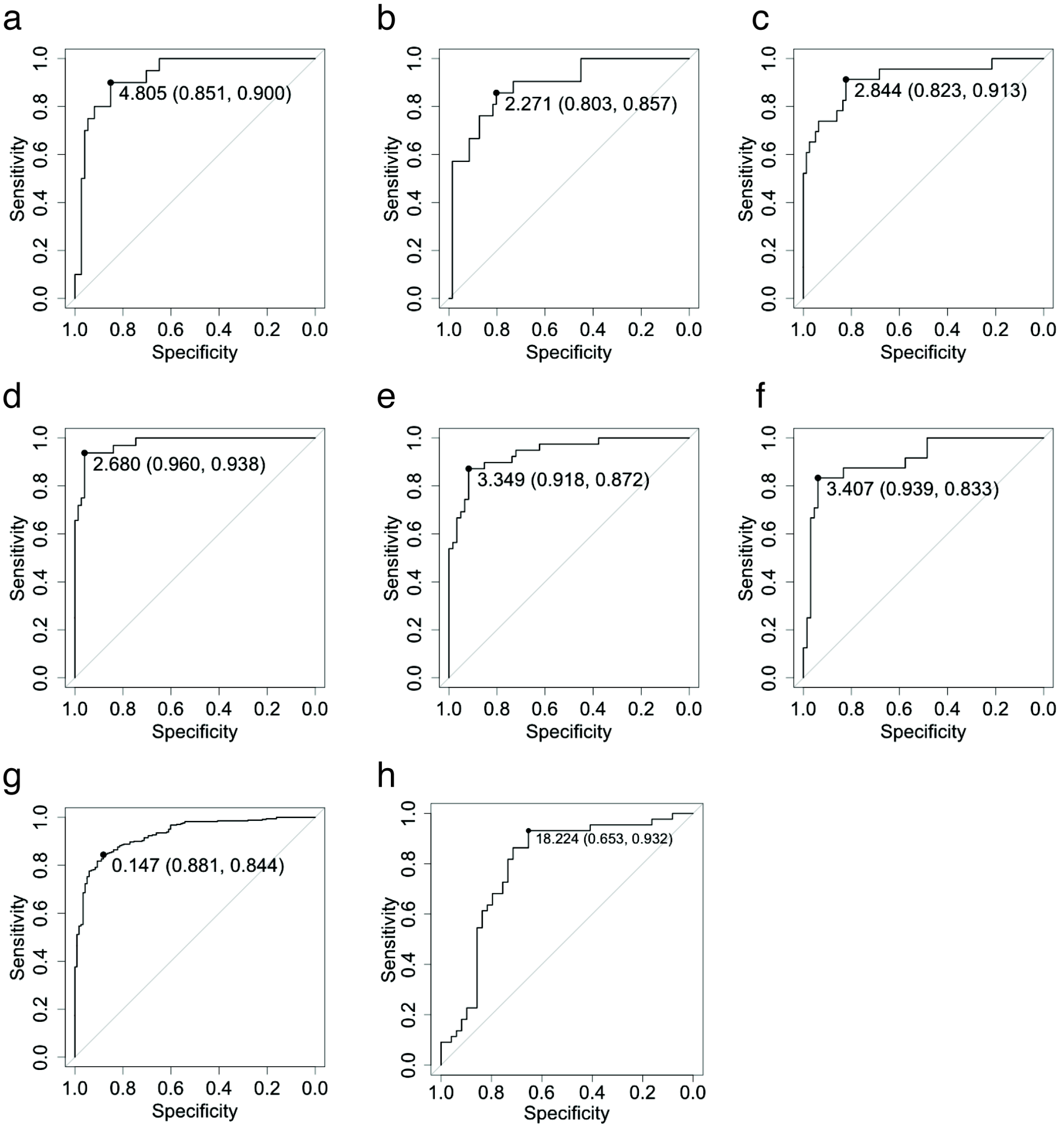

Supplementary Figure S2: Assay-specific Receiver Operating Characteristic (ROC) curves. Indicated curve positions represent thresholds selected for lowest total error rate (FPR+FNR). The numbers in parentheses indicate corresponding sensitivity and specificity, respectfully. (**a-f**) ROC curves for detecting tracking failures in each assay. We randomly selected cells and verified their tracking

accuracy as “pass” or “fail”. These served as ground truth and were used to construct ROC curves across choice of TrAM threshold. (**a**-**c**) Motility assays based on tracking data sets extracted from (**a**) Harmony, AUC = 0.93, $CI_{95\%}$ = [0.87,0.98], (**b**) Imaris, AUC = 0.89 95%CI=[0.81-0.97], (**c**) CellProfiler, AUC = 0.92, $CI_{95\%}$ = [0.84,0.99]. (**d**) Protein translocation assay from Harmony data, AUC = 0.98, $CI_{95\%}$ = [0.96, 1] (**e**) Phototoxicity assay from Harmony data, AUC = 0.94, $CI_{95\%}$ = [0.89, 0.98]. (**f**) Mitosis assay from Harmony data, AUC = 0.91, $CI_{95\%}$ = [0.84,0.98]. (**g**) ROC curve to detect AR translocation and exclude non-responding cells from Harmony data based on $GFP_{nuc/cyto}$ intensity change, AUC = 0.9287, $CI_{95\%}$ = [0.9043, 0.953]. Curve was generated based on distributions of nuclear to cytoplasmic GFP intensity ratio change of ligand treated vs. mock-treated clonal cell. (**h**) ROC curve to detect mitotic nuclei from Harmony data based on max. $GFP_{nuc/cyto}$ change, AUC = 0.7941, $CI_{95\%}$ = [0.6972, 0.8909]. Randomly selected cells annotated as “yes” or “no” for mitotic state to serve as ground truth.

Application of different TrAM thresholds along the curve

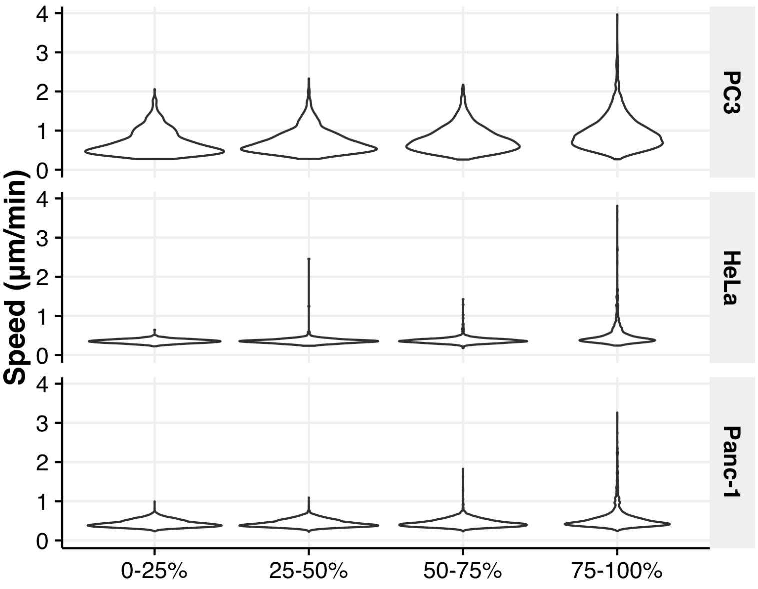

Supplementary Figure S3: Application of different TrAM thresholds along the curve to analyze heterogeneity of cell speed. Thresholds chosen as quartiles and applied to 3 cell lines. The highest sensitivity threshold 1.35 reduced throughput to 25%, 1.84 reduced to 50%, 2.93 captured 75% of the population and 50.44 preserved 100% of the cells.

Impact of cell density fluctuations on Panc-1 cell speed

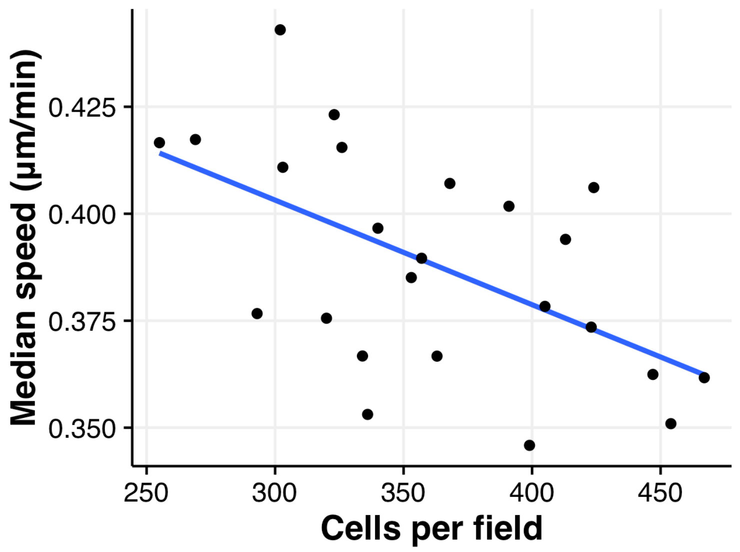

Supplementary Figure S4: Adjustment of Panc-1 cell motility to high density imaging areas (p = 0.005, R2 = 0.31). Plot correlates number of cells per imaging field of with current speed. Each dot represents an imaging well of a 96-well plate.

Segmentation into subcellular compartments

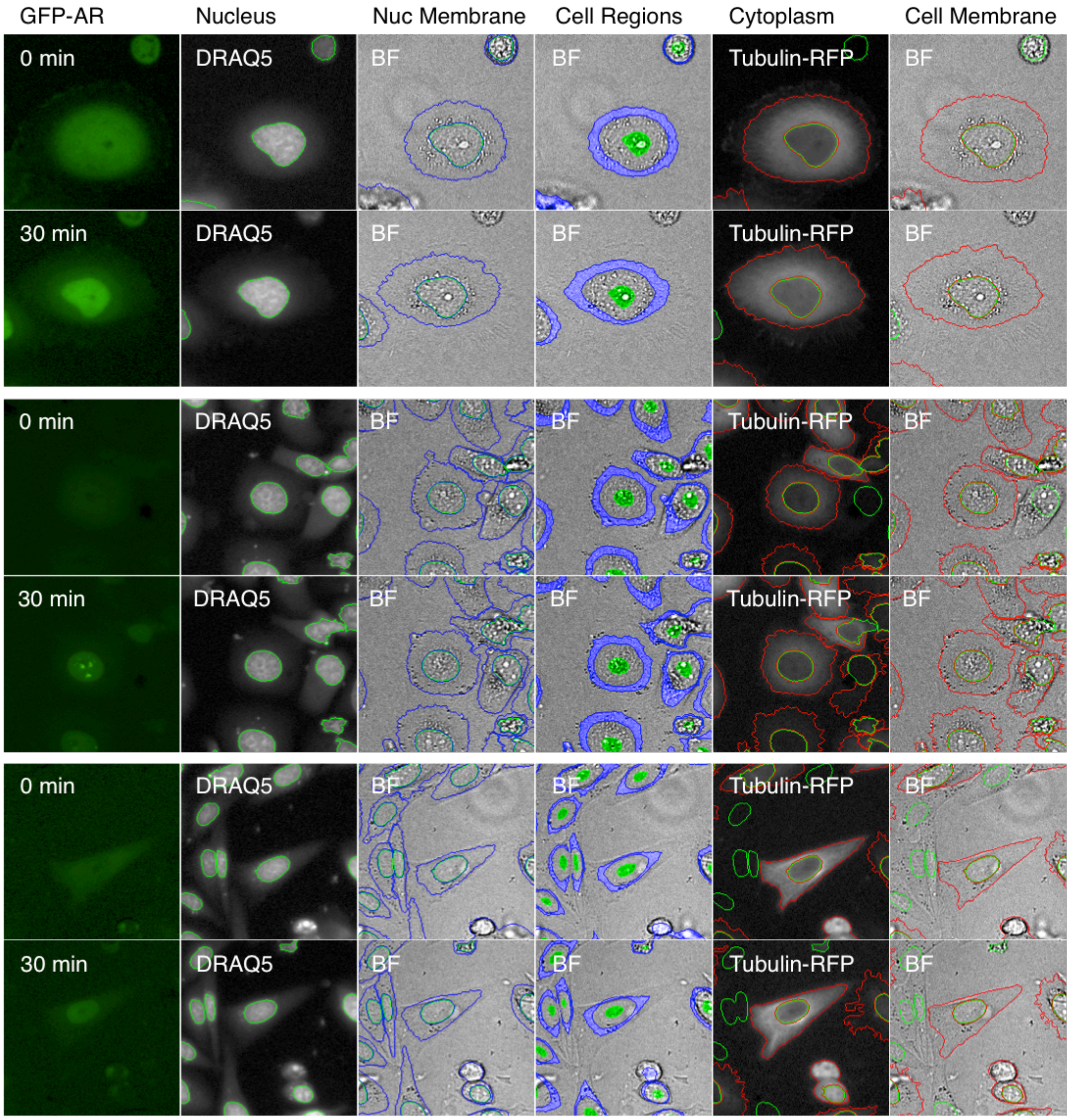

Supplementary Figure S5: Segmentation of PC3 (*upper panel*), HeLa (*middle panel*) and Panc-1 (*lower panel*) nuclear membrane based on DRAQ5 (green outline) and generation of cell regions (outer region purple, inner region green) to measure protein dynamics across nuclear membrane. Cell membrane segmentation based on tubulin-expression (red outline) for cytoplasm

morphology analysis. Segmentation images at baseline (before R1881 treatment) and after 30 min R1881 treatment are shown.

AR translocation in clonal PC3 cell lines overexpressing GFP-AR.

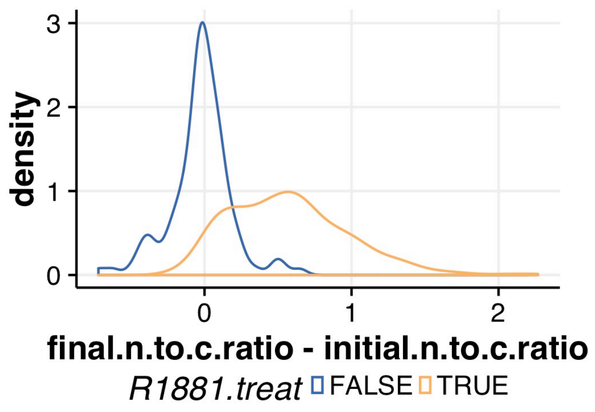

Supplementary Figure S6: Distributions of nuclear to cytoplasmic GFP intensity ratio change of ligand treated vs. mock-treated clonal PC3 cell line expressing GFP-AR (K22).

Application of different K values to identify subpopulations of phototoxic cells

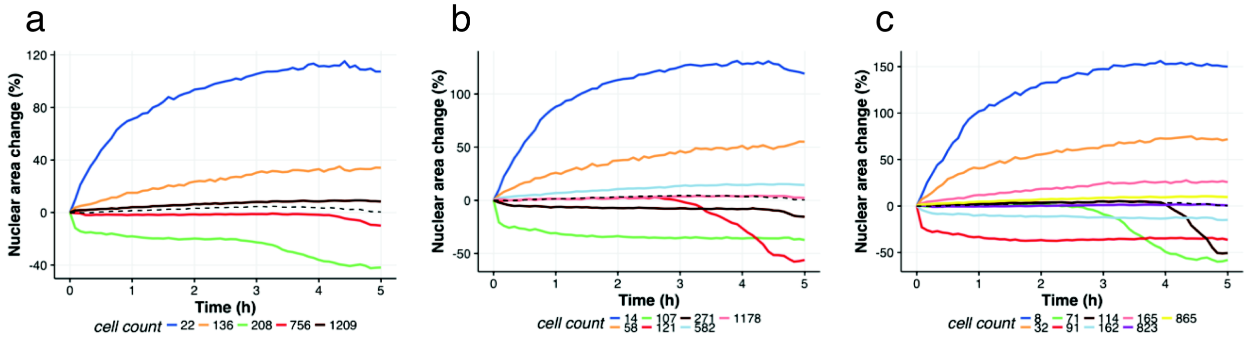

Supplementary Figure S7: K-means clustering of TrAM filtered HeLa cells stained with nuclear DRAQ5 into (**a**) 5, (**b**) 7 and (**c**) 9 subpopulations of distinct nuclear morphology trajectories. Dotted line represents population average.

Optimization of long-term image acquisition: phototoxicity assay of cells expressing Nucleus-RFP

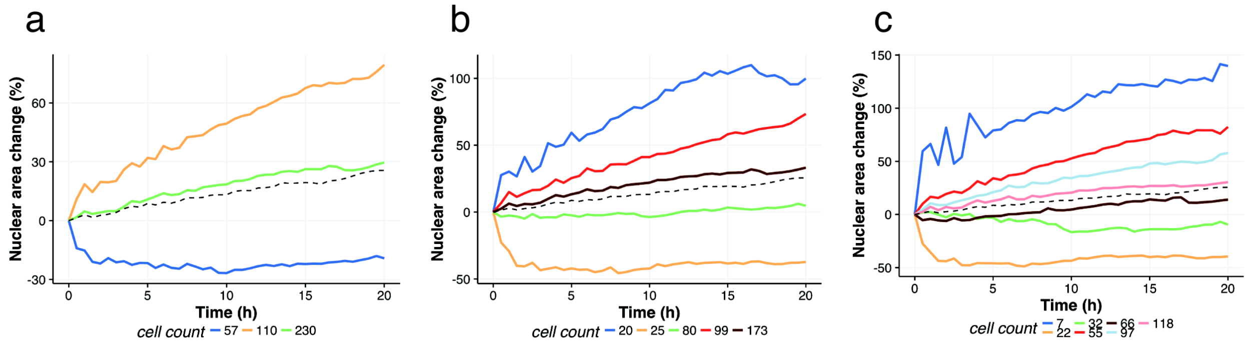

Supplementary Figure S8: K-means clustering of filtered Nucleus-RFP expressing cells into (**a**) 3, (**b**) 5 and (**c**) 7 subpopulations of distinct nuclear morphology trajectories. Dotted line represents population average.

Inhibition of cell proliferation in extended time-lapse experiments of DRAQ5 stained nuclei

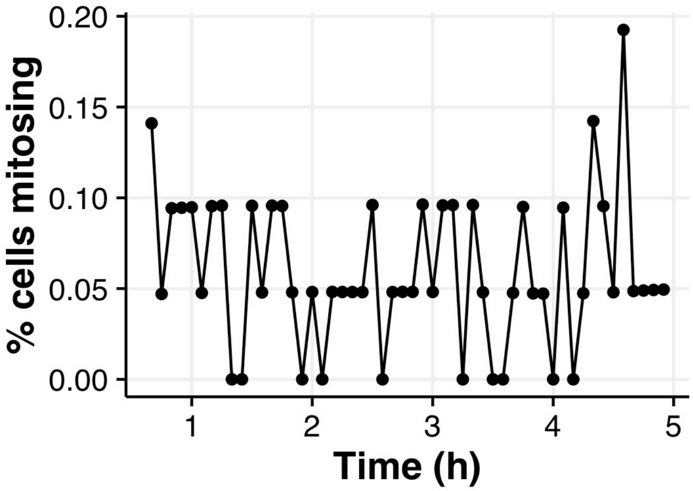

Supplementary Figure S9: Fraction of DRAQ5 stained nuclei dividing over the time course. Imaging increment every 5 min for 5 h.

Tracking HeLa cell phenotypes in response to drug treatments

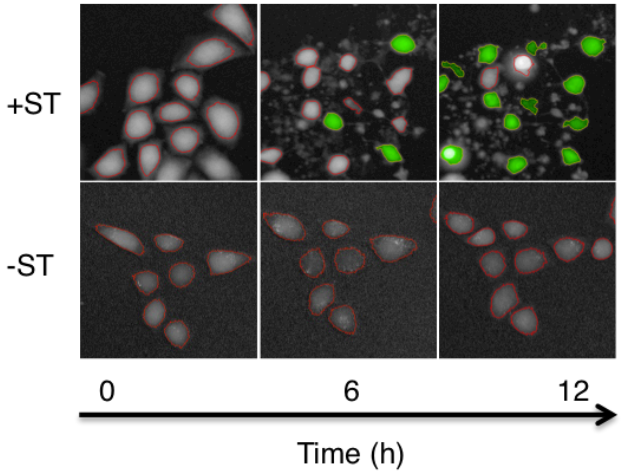

Supplementary Figure 10: Tracking drug responses in HeLa cells using Cell Tracker Orange fluorescent dye. Example images of cells treated with staurosporine (ST) vs. mock-treated cells 0 h, 6 h and 12 h post-treatment. Cell segmentation borders in red, overlay with Cell Tracker Orange. Cells Selected Dead based on Cell Event Green fluorescent dye shown in green.

Evaluation of cell phenotypes in response to drug treatments

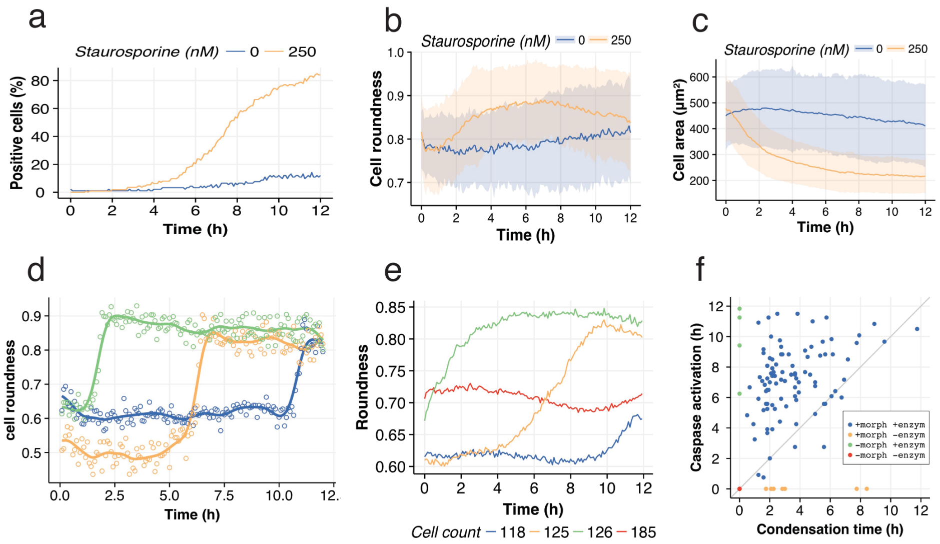

Supplementary Figure 11: Tracking cells from drug treatment to response. (**a**) Fraction of cells undergoing caspase activated apoptosis in staurosporine treated vs. non-treated cell populations. (**b**) Cell roundness and (**c**) cell area plotted against time in staurosporine treated vs. mock-treated cells. (**d**) Single HeLa cells rounding up in response to paclitaxel treatment. Cell roundness plotted against

time. (**e**) K-means clustering of HeLa population into 4 populations of distinct morphological response to PTX, n = 540 cells. (**f**) Correlation of morphological and enzymatic cell response to staurosporine, n = 116 cells. Cell condensation time plotted against time of caspase activation. Colors represent both morphological and enzymatic response (+morph +enzyme), morphological response only (+morph -enzyme), enzymatic reponse only (+morph -enzyme), or no measurable response (-morph -enzyme).

SUPPLEMENTARY MOVIES

Supplementary Movie S1: PC3 cell tracking movie based on Harmony data. Nuclear stain DRAQ5 in gray overlayed with red outlines of segmented nuclei and dots representing XY locations. First frame includes unique cell ID.

Supplementary Movie S2: PC3 cell tracking movie based on Imaris data. Nuclear stain DRAQ5 in gray overlayed with segmentated nuclei in red. First frame includes unique cell ID.

Supplementary Movie S3: PC3 cell tracking movie based on CellProfiler data. Nuclear stain DRAQ5 in gray overlayed with red outlines of segmentaed nuclei. First frame includes unique cell ID.

Supplementary Movie S4: Tracking protein translocation upon ligand stimulation in polyclonal cell line expressing GFP-AR. GFP-AR in gray overlayed with pink dots at nuclear locations determined by Harmony using DRAQ5 nuclear stain. First frame includes unique cell ID.

Supplementary Movie S5: Tracking protein translocation upon ligand stimulation in clonal cell line (K22) expressing GFP-AR. GFP-AR in gray overlayed with pink dots at nuclear locations determined by Harmony using DRAQ5 nuclear stain. First frame includes unique cell ID.

SUPPLEMENTARY DATA

Supplementary Data S1: Tracking data from Harmony, Imaris and CellProfiler examined by eye to generate the ground truth. To generate ROC curves, randomly chosen well and poorly tracked cells (cell.id) were annotated as "pass" or "fail" in the nuc.by.eye.ROC, columns and associated with respective TrAM values listed in the adjacent columns. To estimate tracking failure prevalence, randomly chosen cell tracks (cell.id) were annotated as "pass" or "fail" in the columns nuc.by.eye.total. For phototoxicity and mitosis data, events were additionally annotated as "TRUE" or "FALSE" in the phototoxicity.by.eye and in

the mitosis.by.eye columns, respectively. For the AR translocation assay, cells expressing GFP-AR from a polyclonal population (PC3 GFP-AR) and from a clonal population (K22) were annotated as “TRUE” or “FALSE” for protein translocation in the responder.by.eye column and associated with the calculated $GFP_{nuc/cyto}$ change.

SUPPLEMENTARY APPLICATIONS

Tracking Cell Morphology and Drug Response:

Changes in cell motility and/or morphology can reflect drug response and are thus key read-outs in drug screening assays. Therefore, we sought to develop an imaging, segmentation, and filtering workflow that would track single cells from the time of drug treatment until detectable response.

Image Acquisition. First, we acquired images for 12 h at 5 min increments including a baseline image before drug treatment.

Phenotype Tracking. In these experiments it was important to consider what type of cell label to use: Nuclear dyes don’t provide information on cell morphology and can quickly lead to cytotoxic effects, as demonstrated in **Figure 4a**. Fluorophore-tagged protein expression, e.g. tubulin-RFP, provides highly

accurate cell morphology information but requires filtering of non-expressing cells, thereby reducing assay throughput. Cell Tracker dyes are non-toxic, membrane-permeant, and generally label cells with uniform intensity and provide robust measurements of the whole cell population. Therefore, we performed experiments to track cellular phenotype based on Cell Tracker Orange. We chose a robust algorithm that was adaptable to cell populations with high variation in size and intensity, a prerequisite for tracking the broad morphology changes in response to drugs. We then used these training data sets to adapt the image analysis pipeline to different cell and drug types (**Supplementary Table S4)**.

Data Filtering. Finally, we applied downstream filters to obtain more reliable single cell tracks. Details on criteria used to calculate TrAM and applied thresholds are described in the **Supplementary Information** and summarized in **Supplementary Table S7**. Below we demonstrate an application of the optimized workflow.

Cell condensation and initiation of apoptosis in response to staurosporine: Staurosporine has previously been reported to induce cytoplasmic condensation and both caspase dependent and independent apoptosis[1,2]. We therefore applied the above assays to track heterogeneity of the dynamic phenotype in response to staurosporine.

We performed experiments as described above, imaging Cell Tracker Orange stained cells every 5 min for 12 h, to quantify the heterogeneity of treatment-induced morphological response and the initiation of apoptosis using the CellEvent™ Caspase-3/7 Green Detection Reagent to detect activation of effector caspases in real time. After initial cell tracking based on Cell Tracker Orange, we applied additional image processing to generate cell and background regions that we used to calculate the cell-specific EGFP signal-to-background ratio at all time points (**Supplementary Table S5**). We flagged cells having ratio > 1.1 at any time point as responders having activated caspases and initiated apoptosis (**Supplementary Fig. S10**). Finally, we subjected the preliminary tracking data to downstream TrAM filtering described in the **Supplementary Information** and summarized in **Supplementary Table S7**.

We first evaluated initiation of apoptosis by caspases on the population level. Treated cells had a continuous increase in the number of responders during the course of the experiment, reaching 84% of the population at 12 h. In contrast, mock-treated cells only attained a 12% response rate (**Supplementary Fig. 11a**). We additionally observed morphological responses. Rounding of treated cells occurred, peaking at 6 h followed by cell disintegration (**Supplementary Fig. 10, Supplementary Fig. S11b**). Cells responded prominently to staurosporine by condensation, as measured by an average reduction in cell area by 55%, compared to 8% in the untreated population (**Supplementary Fig.**

11c). Finally, having independently tracked morphological and enzymatic drug response, we asked whether these mechanisms were correlated. In the bulk population, we found a correlation between morphological (defined as > 40% reduction in cell area) and enzymatic response: 85% of cells underwent condensation and apoptosis during the 12 h time course and 4% did not respond either way (**Supplementary Fig. 11f**). Of the remaining 11%, 3% responded morphologically but never activated caspases, and 8% activated caspases but did not condense. Interestingly, cells with delayed condensation were more likely to undergo apoptosis later in the experiment, suggesting that cell morphology, in most cells, predicts cell death in response to staurosporine treatment. To understand the biological significance of these subpopulations and the interaction of these two response mechanisms will require further investigation. Taken together, results from our dynamic drug response phenotyping assay shows that we can identify temporal relationships between phenotypic response mechanisms and response heterogeneity across cells.

SUPPLEMENTARY METHODS

Assay-specific validation points:

Biological Applications: Motility. Fluctuations of cell density have been reported to impact cell speed[3]. In our experiments we detected increased average motility in higher density imaging fields of PC3 cells (**Fig. 2c**). We also validated

significantly increased Panc-1 vs. HeLa cell motility shown in **Figure 2e** by evaluating the cell speed of 166 well-tracked cells (67 HeLa and 99 Panc-1 cells).

Biological Applications: Protein Translocation. Cells overexpressing GFP-labeled androgen receptor are known to translocate the protein to the nucleus within minutes of treatment with the agonist R1881[4,5]. We analyzed multiple PC3 cell lines and measured rapid AR translocation within 30 min, whereas no change in localization was detected in mock-treated cells or cells expressing GFP (**Fig. 3a**). We also validated that responding cells from the clonal and polyclonal cell lines translocated AR at comparable rates by manual assessment of 97 well-tracked responding cells (44 clonal and 53 polyclonal PC3 GFP-AR cells, **Supplementary Data S1**).

Biological Applications: Phototoxicity. The nuclear stain DRAQ5 is a compound known to induce cytotoxic effects in a time-dependent manner[6]. Nuclear condensation is a well-established indicator for phototoxic imaging conditions and an early marker for apoptosis[7,8]. Using DRAQ5 for extended time-lapse experiments and population clustering based on nuclear morphology, we detected condensing nuclei reflective of early phototoxic events (**Fig. 4b**). We validated the fraction of condensing cells detected through our assay (19%) by evaluating 100 cells by eye (17 % identified as phototoxic events, **Supplementary Data S1**).

Biological Applications: Mitosis. Nuclear area has been shown to stably increase during interphase, followed by an episode of nuclear swelling an hour before cell division[9,10]. Analysis of G2 phase duration in HeLa cells (1 h, estimated via nuclear area change, **Fig. 4 d+e**) were comparable to previous reports[11].

Biological Applications: Drug Response – staurosporine. Staurosprine is a potent drug known to initiate both caspase dependent and independent apoptosis within hours of treatment[1,2]. In our experiments, we verified both cell condensation and caspase-activation after 2 h of treatment with staurosporine (**Supplementary Fig. 11f**).

CellProfiler Setup

IdentifyPrimaryObjects:

Select the input image:Cells

Name the primary objects to be identified:Nuclei

Typical diameter of objects, in pixel units (Min,Max):20,50

Discard objects outside the diameter range?:Yes

Try to merge too small objects with nearby larger objects?:No

Discard objects touching the border of the image?:Yes

Method to distinguish clumped objects:Shape

Method to draw dividing lines between clumped objects:Shape

Size of smoothing filter:10

Suppress local maxima that are closer than this minimum allowed distance:7.0

Speed up by using lower-resolution image to find local maxima?:Yes

Name the outline image:PrimaryOutlines

Fill holes in identified objects?:After both thresholding and declumping

Automatically calculate size of smoothing filter for declumping?:Yes

Automatically calculate minimum allowed distance between local maxima?:Yes

Retain outlines of the identified objects?:Yes

Automatically calculate the threshold using the Otsu method?:Yes

Enter Laplacian of Gaussian threshold:0.5

Automatically calculate the size of objects for the Laplacian of Gaussian filter?:Yes

Enter LoG filter diameter:5.0

Handling of objects if excessive number of objects identified:Continue

Maximum number of objects:500

Threshold setting version:1

Threshold strategy:Adaptive

Thresholding method:Otsu

Select the smoothing method for thresholding:Automatic

Threshold smoothing scale:1.0

Threshold correction factor:1.0

Lower and upper bounds on threshold:0.0,1.0

Approximate fraction of image covered by objects?:0.01

Manual threshold:0.0

Select the measurement to threshold with:None

Select binary image:None

Masking objects:None

Two-class or three-class thresholding?:Two classes

Minimize the weighted variance or the entropy?:Weighted variance

Assign pixels in the middle intensity class to the foreground or the background?:Foreground

Method to calculate adaptive window size:Image size

Size of adaptive window:10

TrackObjects:

Choose a tracking method:Overlap

Select the objects to track:Nuclei

Select object measurement to use for tracking:None

Maximum pixel distance to consider matches:10

Select display option:Color and Number

Save color-coded image?:Yes

Name the output image:TrackedCells

Select the motion model:Both

Number of standard deviations for search radius:3.0

Search radius limit, in pixel units (Min,Max):2.0,10.0

Run the second phase of the LAP algorithm?:Yes

Gap cost:40

Split alternative cost:40

Merge alternative cost:40

Maximum gap displacement, in frames:5

Maximum split score:50

Maximum merge score:50

Maximum gap:5

Filter objects by lifetime?:No

Filter using a minimum lifetime?:Yes

Minimum lifetime:1

Filter using a maximum lifetime?:No

Maximum lifetime:100

MeasureObjectIntensity:

Hidden:1

Select an image to measure:Cells

Select objects to measure:Nuclei

MeasureObjectSizeShape:

Select objects to measure:Nuclei

Calculate the Zernike features?:No

Protocol for cell tracking

	**Find nucleus and Calculate Morphology Properties**					**Select Population**	**Select Population**	**Track Objects and Calculate**<br>1. **Morphology Properties**<br>2. **Kinetic Properties**
Population	Nuclei					Nuclei Selected	Final Nuclei	Tracked Final Nuclei
Cell line	Label/Channel	Method	Diameter [μm]	Splitting Coefficient	Common Threshold	Remove boarder objects	Nuclear area [μm^2]	1. Track Object Division<br>2. Correct Detection Errors<br>3. Discard Single Timepoint Tracks
HeLa	DRAQ5 (short assay)	M	20	0	0.18	✔	120-1000 (short assay)	✔
	Nucleus-RFP (long assay)	M	20	0	0.1	✔	70-600 (long assay)	✔
Panc-1	DRAQ5	M	11	0.14	0.20	✔	40-1000	✔
PC3 GFP-AR	DRAQ5	M	16	0-0.03	0.19-0.40	✔	50-1100	✔

Supplementary Table S1: Image Analysis building blocks applied in Harmony to obtain nuclear tracking data for downstream filtering. Short assay conditions were used in motility and translocation assays spanning minutes, long assay conditions for phototoxicity and mitosis assays spanning hours.

Protocol for cell segmentation to measure protein translocation

Harmony Building Block	Find Cytoplasm of Tracked Final Nuclei			Select Cell Region and Calculate EGFP Intensity Properties	Select Cell Region and Calculate EGFP Intensity Properties	Calculate Properties by Formula	Select Population
Output	Cytoplasm			Nucleus Region	Cytoplasm Region	N to C Ratio	Flag Responders 0.9-10
Cell line	Label	Method	Individual Threshold	Resize Nuclear Region [%]	Resize Cytoplasm Region [%]	(Intensity Nucleus Region EGFP Mean) / (Intensity Cytoplasm Region EGFP Mean)	N to C Ratio
HeLa	DRAQ5	D	0.3	Outer border 70<br>Inner border 100	Outer border 10<br>Inner border 30	✔	0.9-10
Panc-1	DRAQ5	D	0.48	Outer border 70<br>Inner border 100	Outer border 10<br>Inner border 30	✔	0.9-10
PC3	DRAQ5	D	0.20-0.31	Outer border 70<br>Inner border 100	Outer border 10<br>Inner border 30	✔	0.9-10

Supplementary Table S2: Image analysis building blocks applied in Harmony to segment nuclear membrane and definition of nuclear-cytoplasmic ring regions to measure nuclear translocation.

Protocol for cytoplasmic segmentation to measure cell morphology

**Harmony Building Block**	**Find Cytoplasm and Calculate**<br>1. **RFP Intensity Properties**<br>2. **Morphology Properties**			**Select Population**
Output	Tracked Final Nuclei			Flag Tubulin
Cell line	Label	Method	Individual Threshold	Intensity Cytoplasm dsRed Mean
HeLa	Tubulin-RFP	D	0.49	> 500
Panc-1	Tubulin-RFP	D	0.37	> 900-1700
PC3	Tubulin-RFP	D	0.37	< 700-2500

Supplementary Table S3: Image Analysis building blocks applied in Harmony to segment cell membrane based on tubulin-expression for cell morphology analysis.

Protocol for cell segmentation to measure cell morphology

Harmony Building Block	Find Cells and Calculate Morphology							Select Population	Select Population	Track Cells and Calculate<br>1. Morphology Properties<br>2. Kinetic Properties
Population	Cells							Cells Selected	Cells Selected Final	Tracked Cells Selected Final
Cell line	Label	Method	Common Threshold	Area [μm^2]	Split Factor	Individual Threshold	Contrast	Remove boarder objects	Filter by property- Cell area [μm^2]	1. Track Object Division<br>2. Correct Detection Errors<br>3. Discard Single Timepoint Tracks
HeLa	Cell Tracker Orange	C	0.3-0.4	> 100	15-18.7	0.1-0.4	0.05-0.1	✔	100-2000	✔
PC3	Cell Tracker Orange	C	0.3	> 30	15	0.1	0.05	✔	80-2000	✔

Supplementary Table S4: Image Analysis building blocks applied in Harmony to track morphological response to drugs in single cells.

Protocol for cell segmentation to measure initiation of caspases

Harmony Building Block	Find Cells	Select Cell Region and Calculate Intensity EGFP Nucleus Region	Select Cell Region and Calculate Intensity EGFP Background Region	Calculate Properties by Formula	Select Population
Output	Tracked Cells Selected Final	Nucleus Region	Background Region	S/B Ratio GFP Mean	Flag Cells Selected Dead EGFP
Cell line	Label	Resize Nucleus Region [μm/px]	Resize Ring Region [μm/px]	(Intensity Nucleus Region EGFP Mean) / (Intensity Background Region EGFP Mean)	Filter by Property-S/B Ratio
HeLa	Cell Tracker Orange	Outer border 0<br>Inner border INF	Outer border -30<br>Inner border -20	✔	> 1.1

Supplementary Table S5: Image Analysis building blocks applied in Harmony to track caspase activation and initiation of apoptosis in real time in response to drugs.

Image acquisition filters

Channel name	excitation	emission	Fluorophore/Stain
DRAQ5	620-640	650-760	DRAQ5
DsRed	520-550	560-630	Tubulin-RFP
			Cell Tracker Orange
EGFP	460-490	500-550	GFP-AR
			Cell Event Green
BF	Transmission	650-760	Label-free control

Supplementary Table S6: Summary of filters used for image acquisition.

TrAM criteria and thresholds

Assay	Parameters	TrAM cut-off and predictive thresholds
Motility	Nuclear area	< 4.69
	Nuclear roundness	
Protein Translocation	XY positions	< 2.68
	Nuclear/cytoplasmic ratio increase	> 0.147
Phototoxicity	XY positions	< 3.35
	Nuclear roundness	
Mitosis	XY positions	< 3.41
	Nuclear roundness	
	Max. nuclear area change	> 18.2%
Drug response: Apoptosis	XY positions	< 4.0
	Signal/Background GFP Intensity	> 1.1

Supplementary Table S7: Summary of TrAM thresholds applied to each assay.

REFERENCES:

1. Ernest, N. J., Habela, C. W. & Sontheimer, H. Cytoplasmic condensation is both necessary and sufficient to induce apoptotic cell death. *Journal of cell science* **121,** 290–7 (2008).
2. Belmokhtar, C. A., Hillion, J. & Ségal-Bendirdjian, E. Staurosporine induces apoptosis through both caspase-dependent and caspase-independent mechanisms. *Oncogene* **20,** 3354–62 (2001).
3. Hartmann-Petersen, R., Walmod, P. S., Berezin, A., Berezin, V. & Bock, E. Individual cell motility studied by time-lapse video recording: influence of experimental conditions. *Cytometry* **40,** 260–70 (2000).
4. Darshan, M. S. *et al.* Taxane-induced blockade to nuclear accumulation of the androgen receptor predicts clinical responses in metastatic prostate cancer. *Cancer Res.* **71,** 6019–6029 (2011).
5. Thadani-Mulero, M., Nanus, D. M. & Giannakakou, P. Androgen receptor on the move: Boarding the microtubule expressway to the nucleus. *Cancer Res.* **72,** 4611–4615 (2012).
6. Smith, P. J. *et al.* Characteristics of a novel deep red/infrared fluorescent cell-permeant DNA probe, DRAQ5, in intact human cells analyzed by flow cytometry, confocal and multiphoton microscopy. *Cytometry* **40,** 280–91 (2000).
7. Bouchier-Hayes, L., Muñoz-Pinedo, C., Connell, S. & Green, D. R.

Measuring apoptosis at the single cell level. *Methods* **44,** 222–8 (2008).

8. Ibuki, Y. & Goto, R. Phototoxicity of benzo[a]pyrene by ultraviolet A irradiation: induction of apoptosis in Jurkat cells. *Environ. Toxicol. Pharmacol.* **11,** 101–109 (2002).

9. Hahn, A. T., Jones, J. T. & Meyer, T. Quantitative analysis of cell cycle phase durations and PC12 differentiation using fluorescent biosensors. *Cell Cycle* **8,** 1044–1052 (2014).

10. van Dierendonck, J. H., Keyzer, R., van de Velde, C. J. & Cornelisse, C. J. Subdivision of S-phase by analysis of nuclear 5-bromodeoxyuridine staining patterns. *Cytometry* **10,** 143–50 (1989).

11. Posakony, J. W., England, J. M. & Attardi, G. Mitochondrial growth and division during the cell cycle in HeLa cells. *J. Cell Biol.* **74,** 468–91 (1977).
